# Data science for pedestrian and high street retailing as a framework for advancing urban informatics to individual scales

**DOI:** 10.1007/s44212-022-00009-x

**Published:** 2022-10-03

**Authors:** Paul M. Torrens

**Affiliations:** Department of Computer Science and Engineering and Center for Urban Science + Progress, Tandon School of Engineering, New York University, New York, USA

**Keywords:** Customer journey, High street, Pedestrian, Spatial behavior, Machine learning, Geographic information science, Big data

## Abstract

**Background:**

In this paper, we consider the applicability of the customer journey framework from retailing as a driver for urban informatics at individual scales within urban science. The customer journey considers shopper experiences in the context of shopping paths, retail service spaces, and touch-points that draw them into contact. Around this framework, retailers have developed sophisticated data science for observation, identification, and measurement of customers in the context of their shopping behavior. This knowledge supports broad data-driven understanding of customer experiences in physical spaces, economic spaces of decision and choice, persuasive spaces of advertising and branding, and inter-personal spaces of customer-staff interaction.

**Method:**

We review the literature on pedestrian and high street retailing, and on urban informatics. We investigate whether the customer journey could be usefully repurposed for urban applications. Specifically, we explore the potential use of the customer journey framework for producing new insight into pedestrian behavior, where a sort of empirical hyperopia has long abounded because data are always in short supply.

**Results:**

Our review addresses how the customer journey might be used as a structure for examining how urban walkers come into contact with the built environment, how people actively and passively sense and perceive ambient city life as they move, how pedestrians make sense of urban context, and how they use this knowledge to build cognition of city streetscapes. Each of these topics has relevance to walking studies specifically, but also to urban science more generally. We consider how retailing might reciprocally benefit from urban science perspectives, especially in extending the reach of retailers' insight beyond store walls, into the retail high streets from which they draw custom.

**Conclusion:**

We conclude that a broad set of theoretical frameworks, data collection schemes, and analytical methodologies that have advanced retail data science closer and closer to individual-level acumen might be usefully applied to accomplish the same in urban informatics. However, we caution that differences between retailers’ and urban scientists’ viewpoints on privacy presents potential controversy.

“As I stumble on past / I see us all in the glass.” (Lorenz et al., 2020)

## Introduction

In this paper, we consider how the idea of the *customer journey*, originally developed for indoor and e-commerce retail settings, might be extended to also consider pedestrian retailing on the high street, and how it may inform urban informatics at individual scale within urban science more generally.

How pedestrians move through built settings, how they reason about them, and how they engage with the environment and other people along the way are key concepts in urban science (Batty, 2013b). Alas, despite their significance, pedestrian phenomena are often challenging to study for at least three main reasons. First, walking exposes people to many different facets of cities, often several at once. The result is that pedestrians’ connections to urban environments while walking take on both shifting and concurrent meaning across many dimensions of urbanity. These include architecture (Turner et al., 2001), urban design (Hess et al., 1999), transportation (Polus et al., 1983), sociality (Whyte, 1980), psychology (Franěk, 2013), computing (Hazas et al., 2004), marketing (Bhargava & Donthu, 1999), environmental science (Hong et al., 2021), public safety (Fyfe, 1991), and public health (Buonanno et al., 2011). Second, at source, pedestrians’ connections to urban life are often uniquely individual. The perception, action, and cognition that pedestrians invoke are formed initially from the independent viewpoint that they have on very local surroundings, which is often dynamically updated in fleeting windows of space and time as they move (Wang & Cutting, 1999). Third, pedestrian behavior scales, in the sense that walkers’ local views can transfer to others in dyads, groups, and crowds (Aral et al., 2009; Barsade, 2002; Hatfield et al., 1993; Laird et al., 1994), and on to larger flows of foot traffic along streetscapes (Batty et al., 1998; Gorrini et al., 2014; Moussaïd et al., 2009). People’s independent experiences on streets therefore form the atoms that make up our broader and holistic understanding of many urban phenomena. Across each of these three factors, empirically studying the microcosm of pedestrian behavior on streets can easily become difficult or even intractable (Allen & Torrens, 2005). Complicating matters, high-fidelity data for pedestrian activity is often in short supply. Most existing data-sets involve small case studies, which makes generalization to laws and norms of urban science challenging to accomplish (Torrens, 2022).

In some ways, Dear (1988) (and later, Soja (1995)) laid the philosophical backdrop for this conundrum more than 30 years ago, when he considered the myriad of possible viewpoints on cities—with millions of inhabitants—as a form of postmodernism. Heavily-populated urban spaces (such as Los Angeles, the focus of Dear and Soja’s exposition) eschew generalization, in the sense that they can only fully be appreciated when deconstructed to reveal the personal vistas of their residents. Recent work in urban computing in many ways attempts to address the difficulty of looking through every lens on city life by *actually* collecting all of the data from all of the lenses in a city (Amaxilatis et al., 2018; McCullough, 2004). This is happening through initiatives to engage in ubiquitous (Weiser, 1991, 1993) or pervasive computing (Saha & Mukherjee, 2003; Satyanarayanan, 2001), for example. In this mode of inquiry, the speedy and tireless abilities of computing to sift through gargantuan mounds of detail are brought to bear in building insight directly from big data (Liu et al., 2022). Those efforts are nascent, and in application to urban informatics much of the field is in the stage of adapting methods from computer science and electrical engineering to handle often unstructured masses of city life.

Yet, many facets of computer science—among them, context-aware computing (Dourish, 2001a; Lin et al., 2014), affective computing (Picard, 2000), and wearable computing (Mann, 1997; Shull et al., 2014; Xia et al., 2019)—directly treat *individual context* as a design factor. Dourish (2001a), for example has framed several components of informatics design as a form of embodiment with one’s surroundings (Dourish, 2001b), i.e., how people experience the physical and social worlds that they encounter through direct and abstract modes of contact that situate them (contextually) in that setting (p. 100). These ideas are not far-flung from Jane Jacobs’s interpretation of walkable cities’ role in vibrant communities (Jacobs, 1961). These parallel developments—in computing, in philosophy of inquiry, and in our unfolding roadmap for urban science—hint that empirical insight could feasibly be polled, individually, from pedestrians as they naturally move and interact in and with urban settings. They also suggest that this sort of scholarship might be tractable at scales that are perhaps broad enough to cover entire downtowns. *In this paper, we explore whether retail data science might provide the framework to advance those ideas*.

Considered collectively, then, across even a small swath of the built environment, we might envisage a massive compilation of pedestrian viewpoints on cities, from which we could begin to weave together a broad tapestry of daily city life from real-time information, perhaps naturally contextualized to the vernacular meaning of pedestrian context. *Indeed, this is a grand challenge that would prove worthy for urban science to tackle*. Alas, much of the information that pedestrians conjure as they walk is fleeting and bespoke to their own thoughts and considerations. Nevertheless, parts of how pedestrian behavior manifests in the tapestry of city life *as data* are actually beginning to become accessible to inquiry, largely due to urban informatics. Consider, for example, that in recent years, the computers and sensors that people carry with them as phones and tablet devices as they walk (Hazas et al., 2004; Sui, 2007), the artifacts of the built environment that they engage with (Batty, 2013a; Townsend, 2013), the increasing omnipresence of cameras mounted to built structures (Hu et al., 2004; McCullough, 2004), and the early introduction of machines with sensorimotor control (of which semi-autonomous vehicles and advanced driver awareness systems are the most prevalent) (Townsend, 2020) have created opportunities for the development of information systems that can sense, and make sense of, pedestrians as they walk. At the same time, the notion that pedestrians themselves might act as remote sensors as they walk is beginning to take shape (Torrens, 2016a; Swan, 2012).

As these developments unfold, we argue, urban science would do well to consider *robust and extensible research frameworks* that can make use of individual-level data from pedestrians in context. This is not always easy. First, there is an almost inherent big data problem in tackling this issue. Second, one is quickly reminded of the postmodernist gaze on individuality and the terrific intricacy of deconstructing urban phenomena into a dizzying array of individual perspectives, formed and ever-reforming in fleeting moments of human experience underneath urban rhythms and motifs. Our contention, in this paper, is that retail data science can suggest some new paths forward. Customer journeys provide a broad scaffold for analyzing shopper movement and behavior relative to retail settings, and the concept of the customer journey has some flexible parallels with ideas about how urban pedestrians move and behave within built environments.

In reviewing the literature, we initially sweep through a high-level overview of walking in urban science. Next, we examine how retailers use the customer journey to frame shopping. This review addresses customer paths, customer experience, retail touchpoints, and the retail servicescape. We then consider potential bridges between retail customer journeys and walking in cities. Next, we review how existing work in urban informatics could be nudged in ways that would allow it to benefit from retail perspectives. This covers pedestrian surveys, movement tracking, tap-ins as touchpoints, data-mining of activities and actions, and computer vision. Following this, we look to possible future paths of research inquiry to align retailing and urban science on the customer journey framework. These topics include the urban omnichannel, data granularity, fast and slow data, high street information systems, profiling and geodemographics, and controlled experimentation. We also examine the pitfalls of privacy and surveillance that circumscribe most discussions of translating retail science to urban applications.

## Pedestrian and crowd dynamics

The act of walking through built settings has long been usefully considered to be a framework for ideas in urban science. Examining how and why people walk through cities is crucial to urban science’s foci on understanding the shifting cadence and patterns of urban movement (Batty, 1997a), individual and group accessibility to resources and to services (Giuliano, 1989; Handy, 1992), the development of urban community (Talen, 1999), the functioning of cities as complex adaptive systems (Batty, 1971), place-based dynamics of environmental exposure and urban public health (Buonanno et al., 2011; Lee & Laefer, 2021; Hong et al., 2021), vulnerability to urban crime (Grubesic & Mack, 2008), the functioning of advanced and often mobile information and telecommunications technologies within cities (Torrens, 2008; Townsend, 2000), micro-economics of locational advantage (Cervero & Kockelman, 1997), and the resiliency of cities as interconnected systems of transportation opportunity (Mishra et al., 2015).

The intertwined relationships between walking and city settings also present as core tenets of other disciplines, with the implication that walking relative to built context could serve as a useful vehicle for cross-disciplinary scholarship. For example, the sometimes controversial (Lauster, 2007) framework of the flâneur has been used to examine urban walking in history and in social theory (Tester, 1994). The meta-field of psychogeography (Arnold, 2019), purposefully relies on walking in the city as a template for building academic connections between geography and psychology. Urban social psychology is perhaps most popularly encapsulated by the work of Goffmann (1963, 1971) and his observational research to evaluate and codify the microcosm of people’s social actions and interactions as they moved or stayed at rest in different outdoor urban design settings. A key element of Goffman’s work that is of relevance to our review here was his team’s use of individual people’s supposed or expected movement through built features as the basis for building sociological theory. Similar approaches were later used by Newman (1972) and Stark et al. (1974) to build empirically-rooted theories of the sociology of anti-social behavior relative to the built environment, and by McPhail and Wohlstein to frame the sociology of collective behavior (McPhail & Miller, 1973; Wohlstein & McPhail, 1979). Similarly, how people move (or do not move) within small pockets of urban context is at the core of sociological attention to neighborhoods and neighborhood effects (Sampson et al., 2002; O’Brien & Wilson, 2011).

The relationship between walking and urban space is a central theme throughout human geography, particularly in urban geography and behavioral geography (as well as in retail geography). This relationship is well-expressed in Tuan’s (1979) work on how people perceive place as an ensemble of experiences, and Golledge’s (1978, 1987) research to uncover how people form mental/cognitive maps as they walk. Rose et al. (2010) introduced a theoretical basis for examining connections between people and urban geography by considering how buildings affect people’s feelings through notions of space and place. Walking, for example, might straightforwardly be considered to be one of the vehicles by which people assemble their perceptions (as cognitive ensembles) through continual contact with (and reasoning about) urban settings. This is what Thrift (2004) referred to as “movement space”. The idea of walking as a concept for examining perception, action, and cognition is embedded, for example, within Hägerstrand’s (1975) and Pred’s (1981) frameworks for time geography. The idea of rhythmanalysis that was originally introduced by Lefebvre (1992/2004) and was revisited (through data science) by DeLyser and Sui (2012) is, in essence, a mapping of time geography to the individual context of the pedestrian in urban space. Rhythmanalysis and time geography both have associations to non-representational theory as a mechanism for examining how people perform and enact human geography by walking (Thrift, 2008; Waight & Yin, 2021). We note that the theme of urban walking as human geography has enjoyed something of a resurgence in interest of late. This renewed attention to walking has been driven in considerable measure by the insights that are now readily available through walking data collected from mobile devices that individual people carry as they go about their daily activities in cities (Ratti et al., 2006; Eagle et al., 2009; Sevtsuk & Ratti, 2010; Batty, 2003). These include quantitative/computational data cast by devices that rely on geographic positioning systems (GPS) (Raper et al., 2007) Wi-Fi localization (Torrens, 2008; Soundararaj et al., 2020), and cellphone-based localization (Hong et al., 2017; He et al., 2015), but also qualitative data that are accessible via devices that people can use to narrate their own self-reflecting experiences via audio (Anderson, 2004) and pedestrians' ability to use their devices to take photographs (Arnold, 2021) of the things that they encounter. Among the new insights that these approaches have produced are developments in emotional geography to reveal socio-emotional processes of affect in proximity from analysis of walking diary data (Curti et al., 2011; Dawney, 2011; Pile, 2010, 2011) and the relationships between bodies and urban spaces (Valentine, 1999; Longhurst, 2005; Hansen & Philo, 2007; Colls & Evans, 2014).

Discussion of time geography also raises issues of the mode by which people engage in retailing on high streets. In this paper, we predominantly consider pedestrian retailing, i.e., the retail activity engaged in by walking customers. In many cases, particularly in the United States, high streets also support retail access by other modes, particularly “drive through” retailing (Seiders et al., 2000) by which customers directly engage with retail kiosks from their car window, or indirectly shop through curbside pickup (Lapoule, 2014). During the COVID-19 pandemic (Hoekstra & Leeflang, 2020), for example, vehicle-based customer journeys through high streets became increasingly normal (Diebner et al., 2020), while high streets effectively became “contactless” environments for retailing. In this paper, we focus on pedestrian customer journeys, although the extension of the idea to vehicle-based customer journeys is relatively underexplored in the literature.

Walking through urban spaces has also emerged as a significant mechanism in computer science, largely due to the infusion of computing into people’s everyday activities in general terms, as well as owing to the particular usefulness of computing along urban streetscapes. Initially, as Internet and communications technologies (ICTs) moved from the (static) Web to mobile platforms, computing followed suit (Cerf, 2016). In particular, there was fervent activity in computer science to develop mobile analogs of traditional computing schemes, including mobile communications protocols (LaMarca et al., 2004), information systems for managing mobile networks (Ulema et al., 2006) and sensor webs (Balazinska et al., 2007), database schemes for moving objects (Wolfson et al., 1998), mobile software agents (Pham & Karmouch, 1998; Lange & Oshima, 1998), and new forms of computer-human interaction for mobility (Feiner et al., 1997), among others. Adjustments to traditional forms of computing, initially designed to handle mobility, soon gave way to entirely new forms of computing. The development of location-based services (LBS) (Hightower & Borriello, 2001), designed atop location-based (and increasingly now location-*aware*) technologies is of particular relevance to this review paper, as is urban computing (Zheng et al., 2014a). Many of these computing ideas have returned, full-circle, to the issue of pedestrians and the locality of the built context for their journeys, for example, through the concept of the quantified self (Swan, 2012; Hudson-Smith et al., 2020) in the smart city (Townsend, 2013). A next generation of computing, designed de novo with mobility in mind, is now beginning to take shape, including new forms of edge computing (Satyanarayanan, 2017) using sensor-on-chip technology designed to bring machine awareness and cloud computing closer to mobile users as they engage in urban activities (Potdar & Torrens, 2019); pervasive computing (Weiser, 1991) to bridge the gap between centralized and decentralized computing; as well as eXtended Reality (XR) that is narrowing the gap between virtual reality and the tangible experiences of walking and perceptual acts of sensing one’s surroundings in urban settings (Çöltekin et al., 2020; Torrens & Gu, 2021). These developments, initially in applied computing, are in turn leading to close-couplings between urban science and computer science in key areas, including context-aware computing (Dourish, 2001a), affective computing (Picard, 2000), and wearable computing (Mann, 1997), with very close analogs to the qualitative frameworks developed in human geography. Work in critical geography is beginning to take notice, with scholarship designed to uncover evolving forms of geosurveillance (Kitchin, 2015; Swanlund & Schuurman, 2016), in particular.

However, the advantages of a multitude and of a wide breadth of walking-based hypotheses and methodologies for urban science might also be considered a hindrance, because so many factors interplay in generating and influencing urban walking, and because much of what we do while walking is subjective in the insights it reveals. In the remainder of this paper, we will advance an argument that customer journeys—as a form of walking for the *specific purpose of shopping* and through the *single lens of high street retail environments*—might help to hone some of the multiplicity and much of the subjectivity surrounding walking in urban science, particularly for the purposes of allying empirical data to walking journeys.

## The customer journey as a framework for knowledge production in retailing

The field of customer journey analytics emerged from prior scholarship about customer experience (Grewal & Roggeveen, 2020; Verhoef et al., 2009). Many retailers routinely engage in informal and formal study, analysis, and modeling of their service offerings relative to customer experience. These analyses may be performed on the service-side of the customer experience (where retailers often have considerable information), e.g., as dynamic store product maps (Meschtscherjakov et al., 2008), service blueprints (Bitner et al., 2008; Voorhees et al., 2017), or multi-level service design (Patrício et al., 2011). Counterpart analyses on the demand-side of the customer experience include retail customer profiling (Sturari et al., 2016), customer value perception modeling (Rintamäki & Kirves, 2017), and related customer-centric performance indicators (see Underhill (2005, 2009) for an extensive overview), where much of the motivations and actions of the customer are accessible with relatively less information, but may be pieced together by considering “typical customer behavior” (Berendes et al., 2018:, p. 219). Customer journey mapping (Rosenbaum et al., 2017) attempts to reconcile the two: to locate and situate the customer experience as it progresses through service-oriented touchpoints (interactions with displays, encounters with staff, retail transactions, etc.). Increasingly, much of the analysis to populate customer journey maps is being generated from digital data as a by-product of retailers’ own information systems (Santana et al., 2020), through customer use of mobile devices while engaged with service offerings (Kang et al., 2015; Tang, 2019), and through in-store sensing and related smart retail systems (Melià-Seguí et al., 2013) that are increasingly automated (Rai et al., 2011). Customer journey maps may manifest as actual cartographic maps: so-called “heat maps” for example (Rintamäki & Kirves, 2017) that localize customer touchpoints in the servicescape (Underhill, 2005), or they may be considered analytically as part of retail operations, as for example in customer experience modeling (Teixeira et al., 2012), where the customer is considered to be a thread in a holistic retail process.

The reader may notice that in the illustrative examples that we have used above, we refer largely to in-store examples of the retail servicescape. This is emblematic of the current state of practice with regard to customer journey mapping and other analytical treatments of paths within the customer experience. Retailers have relatively little control of the customer experience outdoors, e.g., on high streets, and they often have little in the way of empirical insight there to work with. This relative farsightedness is in spite of the fact that for many urban retailers, a majority of customer traffic comes from the street. In recent years, then, a number of scholars have begun to look at the customer journey outside the store. By far, the greatest volume of work examining the customer journey outside store walls has come from examination of e-commerce. In particular, there is increasing interest in uncovering the aspects of e-commerce platforms that drive customer traffic to stores (Iftikhar et al., 2020). Work on the customer journey as a path with tendrils to *mobile* e-commerce (m-commerce) is particularly relevant here (Tang, 2019), because of the potential for mobility to support hybridized modes of physical and digital customer experiences. For example, when docked to tangible locations or service interactions, m-commerce can be used to build very rich databases of *individual* customer encounters with retail service offerings. These possibilities emerge particularly if customers (or would-be customers) engage with stores’ information systems through mobile applications such as customer loyalty schemes, scanning of goods with Quick Response (QR) codes, use of digital coupons at the point of sale, and so on.

### The customer journey

*Customer journeys* are usually considered to be paths through a retail service system or service space. What might constitute a “path” is quite flexible in definition. Generally, customer paths are framed as conjoined progress through (1) choice- and selection-type maneuvers through decision spaces; (2) embodied locomotion through physical spaces; and (3) virtual movement through cyberspaces. (Together, these three customer journeys form the retail “omnichannel” that can span from Online to tangible patronage, and back again.) The decision journey, for example, may take a customer through retail phases of pre-purchase, purchase, and post-purchase services (i.e., the retail “funnel”), in the same sorts of choice and decision hierarchies that determine pedestrian trips (Torrens, 2004) through trip generation, trip distribution, mode choice, and trip assignment (Louviere et al., 2000). A physical customer journey might involve a customer traversing past a shop front, through the store entry, past product displays, and on to a checkout counter. These procedures are similar to Gibson’s (1950, 1966, 1979) framing of individuals’ environmental perception. A virtual customer journey could begin with a location-based coupon sent to a customer through a store’s Online portal, leading them to a virtual journey past product availability displays on an app-based map of the high street, through a virtual purchase procedure, and on to a physical pick-up location in a tangible store. These types of customer journeys have close allegories with many smart city applications, including spatial pricing schemes for app-driven ride-sharing (Bimpikis et al., 2019), for example. Customer journeys as locomotion through retail high streets are perhaps of particular relevance to urban science because they often take place within broader phenomena of high street pedestrian traffic and crowds, which may be composed of shoppers and would-be shoppers but almost inevitably contains pedestrians traveling for other activities.

### The customer experience

Within retailing, customer journeys are usually regarded as a component of *customer experience*. Generally, retailers consider experiences in terms of patronage of retail facilities and consumption of retail goods. In some cases, retailers aim to provide a generalized customer experience to all customer journeys, e.g., minimizing check-out times at kiosks, or maximizing customer dwell time at particular store fronts. In other situations, retailers may tailor their operations to deliver specific experiences for particular customers, e.g., allowing customers with online orders to pick up goods at dedicated service windows, or enabling VIP customers to access parts of the retail site that are closed to others. In these instances, retailers may use the customer journey to individually infer who their customer is or what type of shopping activity they are engaged in. In supermarkets, for example, convenience shoppers may be persuaded by design to visit “grab and go” type kiosks outside the store. Retailers also may drive retail service decisions relative to an assumption that customers and stores co-create experiences. Co-creation can take effect, for example, via advertising and branding, through layout and design considerations, with merchandising, or using interactions with store staff. Increasingly, customers’ use of (and their journeys through) e-commerce platforms is also coming into focus as part of the experiential co-creation of retail servicescapes.

### Retail touchpoints

One of the main features of the customer journey is that it brings customers into adjacency with the retail servicescape at specific points and instances of contact: what retailers refer to as “*touchpoints*” (Ieva & Ziliani, 2018). Touchpoints may be tangible, as when, for example, a pedestrian walks past a sales display outside a store and inspects the goods, when a store associate hands them a flyer on the street, or when a pedestrian uses a vending machine on the sidewalk. In other cases, touchpoints on the customer journey are virtual, for example when a pedestrian makes use of a software agent on a device to find the best price for a product, or when they rate a retail experience after leaving a store or restaurant. Hybrids of tangible and virtual touchpoints are also increasingly common, as evidenced by tap-based payment systems that make use of near field communication (NFC) technologies (Want, 2011) to provide contactless payment to hurry customers through the end-phase of the journey when checking out, or pedestrians’ use of QR codes to bring-up product marketing and informational material on their handheld devices (Hudson-Smith et al., 2012). In some instances, retail touchpoints and urban touchpoints are one and the same, as in the case of you-are-here maps and information kiosks commonly found in city centers, which serve as waypoints for urban journeys and touchpoints for advertised retailers (Nothegger et al., 2004). In some retail experiences, such as mobile gaming, the touchpoints that form from routine engagement in an urban setting while walking are regarded themselves directly as the customer experience (Niantic Labs, 2016).

### The retail servicescape

In retailing, the idea of the *servicescape* is used to encapsulate the varied geographies of service delivery. Customer journeys, then, are primarily considered to be potential paths that hypothetical or known customers could, should, or did take through a given servicescape. Elements of the servicescape act as levers for service operations that retail providers can use to drive change against the customer journey. Within retail stores, these levers may include the siting and situation of different retail activities such as storage facilities, the shop floor, and checkouts to maximize the effectiveness of customer flow relative to day-to-day store operations; as well as the composition of products and service displays; atmospherics such as aroma, lighting, and color-coding; and layout of marketing material for branding. While indoor aspects of the servicescape are perhaps readily apparent to anybody that has visited a retail store, it is important to recognize that the servicescape also extends beyond the store, into the retail high street outside. Along high streets, several overlapping servicescapes for adjacent retailers may come into conflict. Additionally, we might consider that the retail servicescape must often coexist with other urban servicescapes. The ability for retailers to collect data outside store walls is markedly reduced relative to the insights available to them in-store where, by contrast, retailers have quite broad and far-reaching sensing and observational capabilities. The customer journey idea has seen limited application in outdoor contexts, largely because data are hard to come by. The reader might consider, then, that advances in applying customer journey concepts to urban environments could be useful in expanding the original retail concept outdoors, especially in high street contexts. As we will discuss, traditions of relatively stealthy data capture and profiling that are reasonably common within the private spaces of in-store retailing do not transfer well to the outdoors, where they quickly fall afoul of valid assumptions of public good.

### Integrated approaches

A major benefit of the customer journey approach to framing retail analysis is its ability to unify diverse facets of customer experience, with broad applicability across a range of retail environments, retail sectors, and customer types. Retailers are therefore capable of tracing the customer journey through a very complex pipeline of interactions with store operations, starting with the genesis of a purchase (which may be Online), through to direct engagement with product offerings, through purchase, and on to after-purchase dynamics. As we will discuss later in the paper, recent developments in customer journey information systems (CJIS) (Torrens, 2022) have sought to automate the collation of these data-points on the customer journey for insight generation. The vast majority of progress in development of CJIS is considered for the retail omnichannel, drawing in part from e-commerce analytics platforms that may be extended to m-commerce (Chatzidimitris et al., 2020). Work by Berendes and colleagues, however, has examined what it would take to build CJIS that cover whole retail high streets, potentially with insight down to individual pedestrians (Berendes, 2019; Berendes et al., 2018). This opens up the possibility that CJIS could be extended, in concept, to encompass broader considerations of pedestrian behavior along high streets and streetscapes more generally (Torrens, 2016a).

## Existing data correspondences between retail customer journeys and urban pedestrian journeys

Most research of customer journeys has been considered for indoor shopping (Voorhees et al., 2017). Nevertheless, interest in following customer journeys from the high street and into stores (and vice-versa) is increasing, and the counterfoil of customer behavior along streetscapes beyond store walls has recently drawn considerable attention (Berendes et al., 2018). This perhaps suggests that the customer journey framework is apt for extension to urban science and to urban informatics. Two developments are worth mentioning here as context. First, significant numbers of data points have become available from the retail omnichannel, via the personal devices that people carry with them as they shop. Many of these data points map relatively neatly between the cyberspaces of e-commerce and tangible spaces within retail servicescapes, for example when customers scan a barcode with their phone to check pricing, when they use cell phones to pay via digital wallets, or when customers activate a digital location-based coupon offer tied to localization of their Wi-Fi signal (Souiden et al., 2019). The omnichannel is in essence accessible to customers wherever they go and provides tendrils between in-store journeys and components of the customer experience outside the store. Second, there is a growing appreciation among retailers—especially those with high street store fronts—that customer journeys may be tight-coupled to ambient urban geographies of place (Johnstone, 2012; Clarke & Schmidt, 1995), especially in downtown areas (De Nisco & Warnaby, 2013; Hall, 2008; Hahm et al., 2017, 2019). This also ties retailing success to issues of loss of attractiveness of the city center and suggests that customer journey metrics might be intertwined usefully and productively by retailers with metrics of downtown vitality. There are therefore open opportunities for an exchange of knowledge from urban science into retail operations if we can use the customer journey to meaningfully dock theories and ideas from retailing and urban science. Importantly, at least in concept, that knowledge is possibly accessible at the scale of individual people, with rich ties to their ambient urban context.

In what follows, we discuss five dimensions of the customer journey framework that could, in the near term, provide mutual synergy between urban informatics and retail data science. These include pedestrian surveys, pedestrian movement tracking, analysis of urban transactions through tap-ins, urban activity detection by data-mining, and computer vision on urban scenes. In Section 5, we will additionally discuss seven research themes that could provide significant new insight to urban science, but perhaps with lengthy horizons for research and development. We will highlight the urban omnichannel, issues of data granularity, fast and slow data, high street information systems, profiling and geodemographics, controlled experimentation, and issues of privacy and privacy protection.

### Pedestrian surveys

Individual pedestrians on high streets may be surveyed to assess aspects of their trip-making, activity, and personal factors in their decision-making (e.g., preferences, goals, and habits) (Golledge & Stimson, 1997). Surveys may also be used as a street census, to sample flows of customers and determine the demographic makeup of the high street crowd (UK Ministry of Housing Communities & Local Government, 2018). In some instances, pedestrian diaries (what Millonig and Gartner (2011) refer to as the “time-space budgets technique” of data collection (p. 5)) are used to piece-together the time geography of pedestrian journeys with explanatory factors (Middleton, 2009). Feng et al. (2020) discussed the usefulness of survey data as a *supplement* to other data sources for pedestrian study, particularly in their ability to lend factors of personal experience to other data-sets (p. 8). Hopefully the reader may also envisage that retail survey data can be used similarly to accentuate insight about customers and would-be customers on high streets. Of course, surveys are almost always limited by the fact that high street shoppers may not be able to parsimoniously state or reveal why they are engaging in one particular shopping factor or another (Louviere et al., 2000). Millonig and Gartner (2011) stated this well: “questionnaire survey techniques … provide detailed information concerning route decisions and individual habits, motives and intentions … However, as human behaviour is never fully determined by verbalized structures and people tend to adapt their answers – consciously or subconsciously – to what they expect to be socially desired behaviour … accuracy of the results gathered from questionnaires may suffer.” (p. 5). A perhaps overarching limitation of pedestrian surveys is that they are time-consuming to administer over large samples, may receive low response rates when used in public spaces, and are also subject to errors of recall and other biases.

### Tracking pedestrian movement and flow

Retailers often maintain an interest in siting important operations in geographies that might effectively draw customers’ journeys out from the general flow of people along the sidewalks of a given retail high street and into their stores (or away from competitors’ stores): a concept that they usually term as “foot traffic”. Foot traffic is often a key performance indicator (KPI) in determining the onset of retail operations, including initial premises location and opening hours. Foot traffic fluctuations can also serve as a KPI to drive day-to-day decisions about placement of marketing and arrangement of merchandising, as well as momentary operations such as staffing geography.

There are several commonly accepted corollaries between retail foot traffic and concepts of sidewalk pedestrian flow in urban science. These include connections between foot traffic on streets and vibrant communities (O’Sullivan & Bliss, 2020), livable spaces (Talen, 2002; Cervero, 1998; Cervero & Kockelman, 1997), downtown revitalization (Talen & Jeong, 2019), and even notions of defensible spaces (Newman, 1972, 1996). There is a generally held principle that public (and particularly municipal) sidewalks ought to provide a level of transport service for all pedestrians. This has traditionally been examined through physical attributes of walker flow on high streets. The approach is exemplified by the work of Hess and Moudon (Hess et al., 1999; Moudon et al., 1997), who have examined pedestrian flow as a function of micro-scales of urban design and morphology. Sulis et al. (2018) used smart card payment data, cell phone data, and social media data to build urban metrics (based around pedestrian behavior) to explore Jacobs’s (1961) concept of urban vitality. Sulis et al. (2018) connected measures of urban diversity, place vitality, and pedestrian flow. This analysis was carried out coarsely, looking at London as a whole. Feng et al., 2020 provided a recent and exhaustive review of available data collection options for acquiring pedestrian data (largely considered for outdoor street settings) and possible schemes for overcoming some of the inherent difficulties in building empirical records of public pedestrian behavior. These include methods for measuring the flow characteristics of continuums of pedestrians along streets, movement patterns of pedestrian groups within those flows, and choreography of individual pedestrians.

Retail customer journey analysis of foot traffic is often trained on specific types of paths within the background flow of sidewalk pedestrian traffic. Retailers are keen to identify who, among a crowd of mobile pedestrians, might be a customer or potential customer, and therefore the paths that yield signs of customer traffic are of utmost concern within the broader tapestry of outdoor walkers. By extension, the same approaches could be used to identify different types of walkers for urban science. Ideas from the customer journey, then, might be useful in building finer-scale granularity for traditional flow-type analyses of crowd patterns in urban science. Similarly, data collection schemes from retailing have developed considerable sophistication in their ability to situate customer movement relative to well-bounded areas of interest (AOIs) and points of interest (POIs) in retail servicescapes. Some retail AOIs and POIs overlap with urban AOIs and POIs (Hu et al., 2015), e.g., pedestrianized malls and car parks, which often serve as anchor points for movement (and pedestrian cognition) in a wide range of urban environments (Couclelis et al., 1987).

Retailers are often able to discern *individual* customer journeys in rich context, by matching customer movement to well-specified typologies of customer behavior. For example, work by Millonig & Gartner, 2011 showed that retail high street customers can be segmented into comparison shoppers, convenience shoppers, and hedonistic shoppers, by examining their customer paths. These schemes could be incredibly useful in urban science beyond retail geography, for example, in isolating commuters within rush-hour surges from space-time paths of movement, or distinguishing among outdoor workers, visiting tourists, or local pedestrians. They could also be used for surveillance to pinpoint deviations from “normal” paths and this raises the issue of the very slippery slope between urban science for the public good and urban science that exacerbates existing problems of algorithmic bias.

#### Manual tracking by observation

In many indoor retail operations, there is a tradition of using manual shadowing of individual customers as a scheme for studying their behavior in stores. These schemes are well-covered by Underhill (2005, 2009). Invariably, shadowing involves trailing customers as they shop and then noting their behavior along the customer journey using coded observational analysis. This work is massively time consuming and does not extend easily over wide areas or to large numbers of customers.

Similar observational research is common in urban science. Observation of pedestrians along retail high streets is one of the most reliable methods for building data-sets about dynamics on their streetscapes (and in many ways possibly represents the gold standard, although it has difficulties to effect in practice). For example, Millonig and Gartner (2011) demonstrated a tablet-based tool for examining pedestrian movement in urban outdoor environments by manual tracking, but with additional on-device analysis for determining speed and stopping behavior from those traced paths. A similar scheme was introduced by Griffin et al. (2007), Torrens et al. (2011), and Torrens and Griffin (2013) for *indoor-to-outdoor* tracking, incorporating social factors among tracked people. Two important caveats of these approaches to data-collection are that they are very time-consuming (Millonig and Gartner (2011) followed only 57 people in their study), and that the data are necessarily reliant on the events that unfold on the street while you are observing: one will likely have little to no experimental control over the scenario being observed (Feng et al. (2020) (p.5)).

One might surmise that observation data could also suffer from problems of specificity: few high streets are enough alike that you could make easy generalizations from even a handful of observational studies. Nevertheless, Brown, 1994 noted that findings drawn from pedestrian counts across different forms of (outdoor) retail settings are “remarkably consistent” (even across countries) (p. 550) in supporting four main pillars of customer journeys. These are (1) the influence of magnet or attractor stores (such as department stores) on drawing customer traffic and swaying customer circulation patterns (p. 551); (2) the frequent exchange of customers between stores with compatible trade classes (p. 551) (see Nelson (1958)); (3) the gravitational pull of entry and exit locations to the high street (such as bus stops, car parking facilities, and train stations (p. 551)); and (4) the frictional influence of distance on the propensity to engage in customer journeys of a given length (Brown (1994) commented on a somewhat hard limit of 200 m between retail outlets) (p. 552) (also see Brown (1987)).

#### Journeys revealed by geographic positioning systems

Many medium and large metropolitan planning organizations have long surveyed citizens’ journey behavior using travel diaries. Traditionally, these have been paper-based in questionnaire form. Wolf et al. (2001) introduced a scheme to supplement this with GPS data loggers that can automatically track movement. In their work, paper records were replaced with GPS and telephone check-in interviews. Wolf et al. (2001) found the resulting diary data to be as good in quality as traditional paper-based questionnaires. A number of studies have sought to examine properties of movement patterns directly from GPS data (without associated diary information). For example, Zheng et al. (2009) introduced a scheme for directly building journey data from GPS traces, with several elements that could be useful in building knowledge of journeys in urban settings. Zheng et al. (2009) studied GPS traces for the presence of “trajectories”, which they regarded as sequential GPS locations that a user moves through beyond a specific time threshold. They also explored “stay points” in GPS data, as geographic regions with specific distance, wherein a user remained during a specific time interval with consecutive GPS point data. Finally, they looked at the “location history” of GPS traces of an individual’s movement, as a sequence of stay points that were visited, also noting arrival and departure times. Zheng et al. (2009) introduced a scheme for combining these findings in GPS data into a hierarchical search tree that could link stay points as connected journeys. Ashbrook and Starner (2003) explored the topic of journey analysis using GPS points to perform “user modeling” (determining what a user will do, and where, when, and why they might do so). Their aim was to predict a user’s next activity. Ashbrook and Starner (2003) used clustering to compose groupings of location bundles in GPS databases and purposed these bundles to estimate user journeys and a prediction of likely next path. Krumm and Horvitz (2007) adopted a similar approach in their “predestination” model of truck movement from GPS traces. However, while the underlying GPS data in each of these examples are often of high resolution, the resulting knowledge generated about journeys is relatively coarse in space and time (often at intra-urban resolution). Duives et al. (2019) used a trained Recursive Neural Network (RvNN) to predict crowd movement patterns (in real time) between polygons produced by GPS trace clusters, but at coarse resolution (5 to 10 minute displacements). These new machine-learning approaches with high-resolution are quite innovative. Consider, for example, that work by González et al. (2008) to form “individual mobility patterns” from cell-tower positioning (call detail records) produced journeys that actually had up to ~ 700 km displacements.

In other cases, data are also available from commercial aggregators of user localization data. GPS traces, in these cases, typically become usable by a provider of a software service as part of their terms of agreement when a user accesses that service on a location-aware device. For example, Goldfarb and Tucker (2020) have recently published an analysis of visitation frequency patterns for different retail categories using *SafeGraph* data.

There are now several examples of geographic information systems that are built atop high-resolution individual GPS data traces that have been uploaded by citizen contributors (Elwood, 2008; Goodchild, 2007; Haklay & Weber, 2008; Zook et al., 2010). These sources of volunteered geographic information (VGI) can, in some cases, be accessed when users make their contributions open to public view. Novack et al. (2018) described a system for mining OpenStreetMap (OSM) data and producing routes that are regarded as “pleasant” (green, social, less busy). They used spatial analysis (buffering) to map those indices to street segments. However, Flanagin and Metzger (2008) questioned the credibility of VGI, particularly relative to traditional forms of geographic information that are acquired and published by official agencies, and they pointed out potential gaps between the two in measurement quality. Quattrone et al. (2015) showed that VGI systems with crowdsourcing data (such as OSM) are dominated by a relatively small number of contributors, with results that are therefore vulnerable to a series of biases. Specifically, they argue, there is a high likelihood of geographic bias, because the journeys that are shown in the system are the journeys that this small number of super-contributors make. Haklay (2010) also discussed this geography problem, showing, for example, that OSM data for the United Kingdom had systematic gaps in some rural and lower-income areas. Hecht and Stephens (2014) arrived at similar conclusions for the United States. De Longueville et al. (2010) additionally argued that VGI suffer from problems of vagueness.

#### Journeys revealed by Wi-Fi and cellular data

There are instances in which the devices that customers use may not support GPS (users may also disengage their GPS so as not to be location-tracked). GPS also tend not to function well indoors and retailers (and urban scientists) may be interested in customer journeys that start outside but move inside, and vice-versa. A dedicated line of research has therefore opened-up to examine how customer journeys might be identified by other location-aware Internet and Communications Technologies. Amaxilatis et al. (2018), among many other authors, discussed the utility of smart devices in data collection, noting that people may often bring their devices to hyper-local parts of urban settings where other technical infrastructure may not have been installed (p. 1–2). In particular, the in-built positioning functions of mobile phones have been used to study urban movement, most commonly through retrospective examination of call detail records from telecommunications providers (Frías-Martínez, ﻿Soguero, & Frías-Martínez, 2012; Frías-Martínez, Soto, Virseda, & Frías-Martínez, 2012; Frías-Martínez & Virseda, 2013; Vieira et al., 2010). Millonig and Gartner (2011) discussed the potential for use of cell phone positioning to automate movement tracking of pedestrian journeys along streetscapes. They were rather dismissive of the idea for use at the scale of walking and instead recommended that it be used for other travel modes that take place over larger distances (p. 6). Lee et al. (2013) introduced a scheme to essentially automate shadowing to discern customer journeys at individual level within a shopping mall. They used Wi-Fi fingerprinting (isolating the hardware address of a customer’s Wi-Fi chip and tracking its position through triangulation with Wi-Fi access points that it communicates with) to build traces of customer journeys, which they then allied to activity classes based on mall zone geography. In urban science, similar themes were discussed by Torrens (2008). Feng et al. (2020) commented that studying movement via wireless technologies opens up problems of signal strength and the inherent geography of broadcasting devices.

#### Journeys revealed by points of interest and areas of interest from retail review data

Due in large part to the blurring of boundaries between e-commerce, m-commerce, and tangible retailing, there has been considerable research effort invested into connections between traces of people’s journeys through social media and the commensurate overlap with their real-world activity. Much of this work is sourced in analysis of point-of-sale data (Antczak & Weron, 2019), tying transactions to people and locations. Early work in this area extended geodemographics to the point of sale, and was usually based on proprietary customer record data linked within retailers’ internal information systems, which included internal metrics for customer satisfaction. Many retailers now present aspects of their customer experience data in a public-facing manner, and this includes user rating data. Intermediary companies such as *Google Reviews*, *Yelp*, and *Meituan-Dianping* have emerged as brokers of customer review data, for example, and there has been considerable work to tie these data to AOI and POI locations to yield place-attraction data and activity maps (McKenzie et al., 2013; Yang & Ai, 2018). Some work has been done to build journey data using these AOI and POI anchors. Although the journeys are usually coarse in their space-time representation, trip paths can straightforwardly be extracted automatically from such data. McKenzie, Janowicz, Gao, Yang, and Hu (2015) referred to POI data as “geosocial”, combining aspects of social geography with the specificity of locations that may be straightforwardly derived from location-aware technologies or location-based services. Gao, Ma, et al. (2013) have suggested that such systems might form the basis for place-based (“platial”) GIS.

POI and associated review data are generally quite available for restaurant establishments, in particular, because reviews of food and service are closely associated to the restaurant location. Wu et al. (2021), for example, was able to examine the rise and fall of restaurants in Beijing at city-wide scale from POI data. Chen, Chen, and Chen (2017) examined connections between the location traces of volunteers and retail POI locations at regional scale. Using a variant of the Geographic Exposure Modeling (GEM) methodology (Beyea, 1999), Kirchner et al. (2014) surveyed 550 people over 8 months, who agreed to share their mobility data, and connected it to a nationwide POI density map of retail outlets across the United States. Kirchner and colleagues have since extended the concept (Cantrell, Ganz, et al., 2015; Cantrell et al., 2013; Cantrell, Anesetti-Rothermel, et al., 2015), tying urban public health (tobacco use, in particular) to point of sale data, as well as to exposure to marketing material and signs as pedestrians move along retail high streets. Xu (2021) examined data from 8524 hotel POIs in Hangzhou, China and analyzed their coarse-scale geographic clustering. Zhang et al. (2021) have recently mapped connections between culture and patronage at food establishments across five provinces of mid-Eastern China (covering 382 million people), using ~ 2.3 million POI records (from *AMap* data archives) of restaurants. The results are coarse, but the investigative reach of the underlying data is impressive. (A larger study is reported in Jiang et al., (2021)). Lin et al. (2018) examined ~ 72,000 POI records in Guangzhou, China for varying retail categories (building materials, clothing and textiles, convenience stores, grocery, malls, specialty shops, supermarkets) and assessed their street-level centrality (Sevtsuk & Mekonnen, 2012) using a variant of space syntax (Penn, 2003). Their results showed that malls and convenience outlets rely heavily on proximity to central streets (streets with a lot of connections to other streets), while building materials suppliers, clothing retailers, specialty shops, and supermarkets favor “betweenness” (i.e., connections to each other). Han et al. (2019) also used POI data to examine connections between street and road structure and coarse patterns of retail geography (in Zhengzhou, China). Liao et al. (2021) combined POI data with smart card data from public transport to study connections between geographic centrality and retail store location (in Beijing, China), using similar measures of betweenness and closeness, but additionally considering temporal dynamics. The temporal dynamics of POIs for broad retail categories are also well-explored in McKenzie, Janowicz, Gao, and Gong (2015).

For the most part, work derived from Online review-based data is (1) regionally focused, and (2) targeted at revealing general patterns of urban geography, which may be associated with retailing. This is, however, beginning to change, as new databases (e.g., the (commercial) *SafeGraph Places* dataset) are becoming available at high resolution, with reach to hyper-local locations within cities and with the granularity of individual establishments that fall within broad AOIs and POIs. In the data-mining and knowledge discovery literature, much of the focus in investigating AOIs and POIs is tied to predicting movement, particularly the likelihood that aggregate flow data might be reconstructed by building paths between POI centroids (Santos et al., 2019; Chen et al., 2013). It is therefore plausible that POI data (perhaps crawled from social media) could be used to automatically generate candidate customer journey paths.

#### Journeys revealed by geo-referenced social media data

Other work has sought to delve deeply into social media data directly. For example, a relatively large number of papers have been published on the issue of studying movement records in geo-referenced social media data (Frías-Martínez, Soto, Hohwald, & Frías-Martínez, 2012). For example, Jurdak et al. (2015) examined over 6 million geotagged *Twitter* records in Australia, from which they were able to build coarse-scale proxy movement patterns of individual users. Kurashima et al. (2010) introduced a scheme for determining possible travel connections by route generation from sets of geotagged *Flickr* images. Specifically, they used data-mining (cluster detection by mean-shift, followed by Markov and topic modeling) to estimate the probability of associating photographs with visits to particular landmarks. They tested the method on the data of ~ 72,000 *Flickr* users in the United States. Chen et al. (2015) studied movement as represented in geo-tagged social media posts on *Weibo*. Their approach involved associating locations to geographically-coincident keywords POIs in *Weibo* data.

Social media analytics of this sort are popular, because sample data are easy to come by. However, there are known problems with analyses of these data. In particular, the journey distances that are extracted are usually relatively large (often inter-city or intra-urban at highest resolution). Realistically, the paths that are resolved from social media data represent *displacements* in space and time and not movement: indeed, relatively little is known about the actual travel that is undertaken within a displacement. Paths are commonly represented in as-the-crow-flies straight lines, which in many examples (such as the New York City analysis of *Twitter* data by Wang and Taylor (2016)) are at odds with the actual street patterns available for movement. Chen et al. (2015) raise several other salient criticisms. They argue that traditional approaches to data-mining geo-referenced media suffer problems of sparseness and irregularity, as well as the presence of problematic volumes of unreasonable records in the data-sets. Naaman et al. (2012) found that social media data are too sparse to cover even diurnal activity in any but a few cities. The movement data that is extracted from social media may be tight-coupled to specificities of the social media type. For example, *Flickr* data are limited to things that people take photographs of and one could easily argue that this will necessarily bias popular places such as landmarks or tourist sites. Johnson et al. (2016) challenged the assumption that social media data have correspondence to local locations at all. For the *Twitter*, *Flickr*, and *Swarm* platforms, they showed that localness only held for 75% of the data. They also showed that localness of social media data varied depending on the sociodemographics of the users as well as their geography, with strong potential for the introduction of biases. They concluded that this can lead to inaccuracies in analysis on data-sets from those platforms. Feng et al. (2020) made the observation that pedestrian journey data is usually lacking in comprehensiveness and that the data often lack records for multiple dimensions of pedestrian behavior with simultaneity. There are also very thorny issues of quality and bias in these data. In his assessment of VGI approaches, Haklay (2010) made a very relevant comment, that questions about “logical accuracy, attribute accuracy, semantic accuracy, and temporal accuracy” are, essentially open problems (p. 700). More than a decade later, we would argue, all of these issues are *still* open.

Despite the criticisms of social media analyses that we discussed above, it is the case that useful components of journeys can be pulled from geo-referenced media databases. Chen et al. (2015), for example, raised an interesting point: they discussed the potential of “movement semantic analysis” (p. 2) as a way to associate likely user activity to stays and moves (movement between consecutive stops). For example, consider the work of Mor et al. (2020), which introduced a method for differentiating among locals and tourists in geotagged *Flickr* photograph data-sets. They were able to extract interesting properties of supposed journeys from the data, including travel time, travel distance, and travel speed. Zhang et al. (2017) also discovered several properties of journeys that can be built from social media data. In utilizing *Twitter* data to build longitudinal travel records, they were able to infer distributions of inter-tweet displacement, length of displacement, duration of displacement, and travel start time. (However, the discovered displacement distances were relatively large and travel between tweets was simply specified as straight-line connections between geo-referenced locations.) These *components* of social media displacement, we argue, could become useful points of further investigation for journey analysis, perhaps at local resolutions that could reach to the scale of high streets.

### Touchpoints and tap-ins

For many of the studies of movement discussed above that are sourced in social media data, there is an implied premise that the act of uploading something to social media repositories (a comment, a photograph, a review) constitutes a touchpoint with the environment. However, a user may have composed the social media data long after they have left the place that it indexes. Consider, for example, the work on bank note tracing presented by Brockman et al. (2006), which tracked inter-city and inter-state displacement based on user tagging of bank note serial numbers. This work is described as revealing “human travel”, but relies on users accessing a Web site to fill out bank note details on forms. One has no idea if this took place at the actual touchpoint of a bank note transaction, or at home when a user has time to look through their currency. Similarly, we mention again that for photograph repositories, people will likely only take photographs of things that they regard as photogenic. They may have many other touchpoints with scenery or the built environment that are mundane and therefore not part of the record. Nov et al. (2008) discussed, for example, how tagging activity on social media may have varied motivation. This implies that tagging can easily be disjointed from behavior and from touchpoint activity. For popular features of the built environment, such as landmarks, social media data may well provide very useful touchpoint data. Kurashima et al. (2010), for example, were able to identify landmarks, camera interests and foci, and inferred routes between landmarks by studying users’ histories on the *Flickr* repository. Zheng et al. (2009) were able to build AOIs from GPS traces. The AOIs and POIs that are routinely extracted in analysis of geo-referenced social media are touchpoints of a sort, *albeit at very coarse scale*. Nevertheless, these could be used as a starting point for fine-grained analysis, especially if we consider that many AOIs and POIs serve as anchor points for movement and possibly for spatial cognition (Couclelis et al., 1987).

Other touchpoints in urban activity match quite closely to retail touchpoints. Consider, for example, that the widening use of tap-in schemes for accessing transportation (buses, trains, bicycle shares, taxi services) has created anchoring schemes for urban journeys that are similar to those for retailing that can be indexed to the point of sale. City transit systems actually deliver customers to high streets in the same way that they ferry them to their jobs and activities (Mishra et al., 2012, 2015). Also, when people tap-in and out of a transit system, they are doing so to pay for their passage. In this way, tap-ins resemble retail data: a customer journey is strongly-typed to a financial transaction and they are also usefully encoded with a cost and therefore a willingness to pay. Batty and colleagues have investigated tap-ins cataloged through transit riders’ use of the *Oyster Card* payment system. In Roth et al. (2011), they leveraged transit tap-in/tap-out ticketing transaction records that note users’ location of entry and exit to the London transit system to examine the city-wide tapestry of transit journeys across London. Their analysis of the *Oyster Card* database showed that trips yield a polycentric spatial pattern in aggregate (likely following the hub model of the transit network itself and the central place theory that it follows), but highly complex patterns in the disaggregate. In Sulis et al. (2018) they tied some of these patterns to measures of urban vitality, using adjacent social media data. In Reades et al. (2016), they examined how the journey data might be useful for managing service disruptions, in essence by examining what we could consider as the servicescape of London’s transit system through the lens of the journeys that it supports. Analyses of the *Oyster Card* database are illustrative of the availability challenges that urban scientists often face in working with data. For example, Reades et al. (2016), in studying the year 2012 records for the database, identified 17.9 million tap-ins and 19 million tap-outs (consider that both events are touchpoints) across 296 stations (p. 372), with 1.65 million tap-ins just during the 8:00 a.m. to 9:00 a.m. morning surge (p. 373). This is a huge amount of data and (automatically) captures a significant facet of Londoners’ daily travel (where they begin and end their transit journey) with precision. But without a connective thread to other data points regarding the individuals that tapped-in or out, we are left with a significant amount of mystique about the journeys that preceded and followed the touchpoint event. Who are the commuters, what were they doing before they entered the transit system, what do they usually do when they leave?

An important overarching issue is that one must of course recognize that subway and bus rides are not walking. The same is true of related analysis of taxi cab (Guo & Karimi, 2017; Castro et al., 2013) and bike share data-sets (Talavera-Garcia et al., 2021). Transit mobility is an accommodation to the problem of mass and collective transport of huge numbers of people; transit trips are therefore very different than customer journeys. In this sense then, perhaps the challenge of collecting massive troves of journey data for pedestrians remains largely unresolved. Indeed, the existing state of the art still points to essentially bespoke case studies designed to collect data (Helbing et al., 2005; Seer et al., 2014). In transit systems, there are also a very limited number of touchpoints (maybe even just one in the case of a bus) that users “check-in” with. If the number of touchpoints between urban mobility data traces and the city could be expanded, we would have a much richer database of actual individual interaction and transaction with the built environment. Journey-spanning information is unlikely to be available within existing municipal databases, save small case study data regarding travel diaries. However, it is conceivable that the data could be collated from multiple sources. Indeed, this is perhaps the lesson that we can learn from retail data on the customer journey. For the outdoors, a variety of firms are in the business of collecting and synthesizing aggregate data, and this is particularly well-known for the science of geodemographics (Longley, 2012), for example. Sulis et al. (2018) have attempted this sort of compounding approach with data that *are* accessible to the public, in their analysis of connections between tap-in data for the London transit system and geo-tags of social media posts from a sample of *Twitter* data streams. They refer to these sources as “mobility data”, but the sense of mobility comes from difficult analysis. Indeed, the raw data comes from static (in space and time) transactions (tap-ins or micro-blog posts) and the mobility must be inferred from those data crumbs. This inference inevitably leads to a network analysis, where mobility is assigned to edge connections between vertices (Mishra et al., 2015). Nevertheless, the work is incredibly promising.

Some researchers have also begun to consider whether retail card transaction data (either sales transactions or customer loyalty transactions) could provide a source of information for customer journeys. Work by Kohsaka (1997), for example, showed that retail trade areas can be automatically derived from analysis of customers’ use of chip-enabled credit cards in the area around Chitose-Karasuyama Station in Tokyo. These transactions, as with tap-ins to transport systems, represent significant touchpoints with retailers that can be isolated to specific locations (usually the point of sale, and the information systems that it connects to) and times as base ingredients for larger algorithmic recipes for journey analysis. Work by Longley and colleagues (Berry & Longley, 2005; Rains & Longley, 2021), for example, has uncovered the potential for deriving micro-geodemographics on loyalty transactions, in particular. This extends the coarse and regional scale idea of geodemographics as a way to segment populations by geography to new, hyper-local specificity. An overarching challenge in pursuing this sort of research, however, stems from the overwhelmingly proprietary nature of most transaction databases. Unlike tap-in data for transit, transactions and loyalty data are usually held by private commercial companies, rather than the municipal agencies that collect transit data.

### Data-mining activities and actions atop social media data

Many retailers make rich use of social media data to assess customer experience activity (particularly post-purchase) to market high streets to consumers (Grewal & Roggeveen, 2020). In essence, this serves to connect customer activity to “lifestyles” that can be associated to those activities. Developers of social media platforms themselves have rich sets of high street data that are produced by their systems, and these data form a core part of their omnichannel advertising (Nield, 2020; Chauhaun, 2019), although much of these data may well be hidden to user view and inaccessible to retailers. Platforms such as *Foursquare*, for example, are quite overt about their reliance on high street data (Frith, 2013), and tying social media marketing to activity.

Researchers in urban science have also mined social media data-sets to try to infer urban activity, as well as actions of urban citizens. The determination of densities of pedestrian activity within intra-urban areas is now routinely explored through analysis of geo-located social media postings and data from social check-in services (Yang et al., 2019). Gabrielli et al. (2013) referred to geo-referenced social media as “opportunistic” data (p.34). In their work, they classified activities from social media data-sets *per location*, which they were able to couple with people arriving at a location or leaving a location. These activities were coarse, borrowing largely from POI classes (arts and entertainment, food, recreation, nightlife, college, shop, etc.), but have broad relevance in connecting retailing and other urban activities. Hasan and Ukkusuri (2014) used topic modeling on geo-referenced *Twitter* data to classify activity patterns as part of their work to examine whether social media could inform activity generation in traditional four-step travel models. The range of activities that they considered were broad: home, work, entertainment, recreation, shopping, and education were among those revealed. Further, they included a wide range of locations and sites for activity, ranging from supermarkets to theaters, libraries, and event spaces (p. 366). A significant result of that work was their ability to isolate *user-specific* activity patterns. However, they cautioned that the activities lacked explanatory power and they pointed out that many activity sequences in the record were incomplete, as well as being over-representative of younger people. Rashidi et al. (2017) showed that social media data over-represent discretionary and leisure activity. Lee et al. (2016) examined ~ 63,000 geo-tagged *Twitter* records across 116 users in Santa Barbara, CA and generated envelope hulls to map their activity space across a weekday/weekend typology. This implies that activity classification from social media could be associated to time geographies (the hulls of Lee et al. (2016) resemble potential path areas, for example), which perhaps hints that activities could be mapped to journey paths and embedded within space-time prisms, should data at sufficient granularity be available. Torrens (2016b), for example, has already shown this with synthetic data produced by agent-based models.

### Examining journeys with image processing and computer vision

Significant progress has been made in automatically extracting movement data from video using computer vision and machine learning schemes (particularly deep learning via multiple layers of artificial neural networks). In some instances, signatures of high street pedestrian flow can be extracted from video scenes, including relationships between characteristics of the streetscape, density of high street occupation, pedestrian traffic volume, and average speed of pedestrians (Krausz & Bauckhage, 2012). Movement characteristics of individual pedestrians or groups of pedestrians (Favaretto, Dihl, Barreto, & Musse, 2016) can also be garnered from video footage (Hoogendoorn et al., 2003), including details on their locomotion (gait characteristics, step frequency, velocity changes) (Hediyeh et al., 2015); preservation of personal space (Moussaïd et al., 2010); and their movement (trails of movement paths and movement events, such as steering maneuvers or stopping behavior) (Moussaïd et al., 2009).

However, sources of video data for high streets can be difficult to come by. Some authors have relied on footage from closed circuit television (CCTV), which often are limited to fixed views on streetscapes from overhead. In other examples, researchers have collected their own data. Several recent video-based experiments have looked into the motion of individuals and crowds within outdoor events (Helbing & Mukerji, 2012; Johansson et al., 2008), and these would seem to resonate with adjacent characteristics of retail high streets during mass events such as celebrations and festivals (Batty et al., 2003a, b). Recent work by Sun et al. (2021) has shown how commercially-available drone platforms can be used to analyze high street movement, in their case to examine the impact of building entrance geometry on the movement patterns of people on the street outside. Methods for machine learning from video could be useful in supporting knowledge-building of urban journeys in two key ways: (1) automated extraction of journey data from video footage of real streetscape scenes, and (2) the performance of streetscape audits to classify and label urban environment scenes in video.

#### Pedestrian tracking in video

Grant and Flynn (2017) presented an overview of the state of the art in pedestrian tracking in video, largely from an image processing perspective. They noted the now wide array of schemes available for scanning video on a per-pixel basis, or on the basis of texture, to extract features of pedestrians and their movement. They also reviewed object-level analysis, including training-free Bayesian clustering to identify independent movement, use of trained support vector machines (SVM) for head tracking, and optical flow for dominant motion extraction. Junior et al. (2010) adopted the same pixel, texture, and object typology in their review. Tripathi et al. (2019) reviewed the range of deep learning schemes (largely using convolutional neural networks (CNNs) that automate machine learning) available for analyzing crowd patterns in video, including crowd counting, estimating the density of crowds, segmentation of patches of activity within crowds, detection and tracking movement, and categorizing crowd behavior into normal and non-normal patterns.

Batty et al. (2003a) introduced an early example of pedestrian analysis in urban science from video. In developing their cellular automata model of carnival crowds, they consulted overhead footage of crowd patterns from helicopters. Hoogendoorn et al. (2003) described an experiment to extract movement traces of pedestrians in crowd motion from overhead video cameras. They examined video footage of 80 student volunteers in an auditorium, using pixel clustering and Kalman filtering on the video to track assigned colored hats that the participants wore as they were directed to walk with assigned behaviors. Johansson et al. (2008) showed that rough characteristics of crowd flow (speed, density) can be extracted from video using straightforward edge and circle detection and tracking. Hussein and Sayed (2019) briefly discussed their scheme for extracting pedestrian movement paths from video, as a step in calibrating pedestrian agent-based models. Using head tracking, Favaretto, Dihl, and Musse (2016) were able to extract very useful quantitative properties of pedestrian motion from overhead video sequences, including position, speed, and angular variation (p. 203); using these features, they were able to build short-lived tracks of individual movement in crowded scenes.

The video-based applications that we discussed above—journey extraction and pedestrian tracking—could help to automate much of the situational awareness, and perhaps also the semantic awareness, necessary to inform analysis of *outdoor* urban scenes using customer journey methodology. Tracing tracks through video provides direct journey data, at the scale of individuals, while scene segmentation allows for the urban streetscape to be blueprinted as if it were a servicescape. Further analysis between the two could support the investigation of urban touchpoints at the level of individual transactions and interactions. Feng et al. (2020) pointed out that most existing video-based studies of pedestrian movement relied upon unrelated third party video data, with the result that it is not possible to control the views and locations of the data (p. 4). For most video studies, only a relatively small window of time (and of space) is covered. In essence, the sampling strategy is relatively arbitrary. Feng et al. (2020) also discussed how the accuracy of movement (and activity) data extracted from video is sensitive to the physical configuration of the camera (angle, resolution, etc.) (p.5). Millonig and Gartner (2011) cautioned that observation by video is often constrained to small areas within view of cameras and that it is reliable only for “loose groups of people” (p. 6). This critique is likely fading in relevance now that machine learning schemes are routinely available to discern individual and very fine-scale features of humans in video (Redmon et al., 2016). Nevertheless, the additional comment by Millonig and Gartner (2011) that blanketing entire sections of an urban area (such as a high street) would require many devices (p. 6) remains an open and thorny challenge.

#### Streetscape auditing in image collections

Static scenes from video footage can also be used to provide individual perspectives on *urban context*. Kelly et al. (2013) described a system for auditing the built environment by inviting human users to study 288 street segment images of St. Louis, MO and Indianapolis, IN on the web archive available via *Google Street View*. The data that form *Street View* are recorded as video by *Google* drivers, but are presented to users as static image mosaics, georeferenced to street centerlines. Kelly et al. (2013) evaluated inter-rater reliability and concluded that *Google Street View* is a viable medium for performing audits. Mooney et al. (2016) used *Google Street View* images to manually associate streetscape features with pedestrian injury using statistical models in New York City. Doersch et al. (2012) examined the question of “what makes Paris look like Paris?” using image processing to extract salient features of Parisian urban form, which prompted a series of investigations into what features of built setting could be extracted from imagery using automation instead of human crowdsourcing. Liu et al. (2016) used image processing to perform automated evaluation of urban environments, focusing on street façade quality and street wall continuity. Their approach relied on scale invariant feature transform (SIFT) (Lowe, 2004), and the *AlexNet* (Krizhevsky et al., 2012) and *GoogLeNet* (Szegedy et al., 2015) CNNs, trained against the *Places* labeled dataset (Zhou et al., 2016). Liu et al. (2011) compared their image processing scheme to ratings provided by 752 passers-by in 56 urban locations. Naik et al. (2014) proposed a system, “Streetscore”, for deep learning on images of streetscapes to assess their relative “safety”. They used training data gathered from 7000 Online participants (tasked with very simple A/B evaluation criteria) to then analyze 1 million images from *Google Street View* across cities in the United States. In later work, Naik et al. (2017) used image processing to look at relative change in appearance of streetscapes through *Google Street View* imagery. They mostly focused on addition or loss of built development in the images. Li et al. (2018) presented a deep learning scheme (image regression by support vector regression) for estimating building age from *Google Street View* images. Their approach extracted features from images, then built a regression model from those features using a CNN. They considered only single-family residential houses and reported 25% error rates, with errors of up to 11 years in estimating building age. Hara et al. (2013) asked human labelers on *Amazon Mechanical Turk* to assess sidewalk accessibility in *Google Street View* images. They found that human labelers were 80% accurate (93% with control measures) in identifying accessibility concerns in images (e.g., no curb ramp, objects blocking access, surface issues, early termination of sidewalks). Law et al. (2017) used machine learning to evaluate the relative quality of street frontage. They used a CNN on *Google Street View* imagery. They examined images for the presence of “active frontage”, e.g., first floor of buildings with windows and doors (as opposed to, say, walls and fences). They referred to their CNN technique as “StreetFrontageNet”, which used United Kingdom Ordnance Survey data to build a graph in which each vertex is a junction and each edge is a street. They also built 3D models of streetscapes using *ESRI City Engine* and programmed an agent to traverse the model via random paths to produce images. Law et al. (2017) concluded that *Google Street View* images are not entirely reliable: they suffer from problems of lighting and obfuscated views.

## Speculative connections between walking in retailing and walking in urban science

In the previous section, we reviewed the literature to draw comparisons between elements of customer journeys from retail perspectives that may have support in existing urban science research that either extends to the individual or gets close. In this section, we look farther afield to explore whether new paths for research inquiry might be usefully opened-up for urban science at individual scale, with the customer journey framework as a guide.

### The urban omnichannel

The retail omnichannel can provide retailers with significant insight into who their customers are, and into the factors that shape their customer journey. This is particularly useful for retailers that have a presence in many different channels (physical stores, e-commerce portals, social media branding, etc.) because aspects of the customer journey such as product comparison may begin in one channel, while the actual act of purchasing and exposure to other aspects of customer experience may complete in another channel. Three conditions of this insight are relevant to our discussion of urban science at individual-scale. First, once developed on cloud, Web, and device platforms, acquisition of omnichannel insight is often automated (Bell et al., 2014). Second, developing empirical connections between omnichannel data points is essentially baked into most omnichannel information systems (Iftikhar et al., 2020). The consequence is that any collected data usually functions as linked data (Wood et al., 2014), with the links providing significant usable structure within and across the data. If a customer can be individually identified in any channel, they can be mapped to activity in the other channels. Third, the two pieces of understanding that we have highlighted (who customers are and how their journey forms) are highly actionable relative to many retail service problems, such that retailers may be able to (automatically) build strong explanatory and semantic connections between customer journeys and the servicescape from those data. Put together, the three factors imply that individuals can be bound to context through automated analysis. For example, Ghose et al. (2019) showed that consideration of customer trajectories in a mall can increase the speed of uptake and redemption probability of mobile advertising, as well driving higher transaction values. Structured analysis across retail channels is often critically important for retailers: as Piotrowicz and Cuthbertson (2014: p. 6) put it, “Because the channels are managed together, the perceived interaction is not with the channel, but with the brand.” For retailers that can effectively manage channel integration, significant efficiencies can be garnered. Chen, Li, and Sun (2017) have shown, for example, that mobile geo-targeting of customers (essentially, sending customers coupons and advertising based on their location while on the move) produces more firm profit than standard forms of targeting. Berendes (2019) made the highly relevant statement that a wealth of digital information from the omnichannel “has created new opportunities to learn more about customer behavior in high streets … While customers in a high street are unknown for the retailers, online retailers are situated with detailed knowledge about their customers” (p. 313). In particular, the fact that many customer journeys now begin their decision journey in e-commerce (comparing goods, assessing stock, checking prices, viewing product information) allows retailers to build large archives and anchors for individual customer journey data before a shopper even sets foot on the high street.

Urban walkers might also be usefully considered to be journeying through an “omnichannel”. Consider, for example, that many walkers now travel with mobile phones that natively track their position (Hightower & Borriello, 2001) and record location histories of many of their transactions either directly through GPS or as part of the telephony system (Hong et al., 2017; He et al., 2015) and the Internet (Torrens, 2008). A significant amount of work in critical geography is revealing how, from the perspectives of these systems, people can be considered to be co-existing across the tangible geographies of their movement through cities and the cybergeographies (Dodge & Kitchin, 2000) of their matching “data shadow” (Clarke, 1994) and “digital twin” (Batty, 2018) in Online realms (Dodge & Kitchin, 2005; Batty, 1997b). Walkers also routinely make use of mobile applications to plan and orchestrate their trips (Mishra et al., 2015), to engage with waypoints along their journey (Ishikawa et al., 2008), and to directly navigate while mobile in cities (Laurier et al., 2016). In doing so, walkers may match and dock significant aspects of their spatial behavior to allied channels of information (and data collection) in the application interface, and increasingly through the larger application ecosystem that mobile software is connected to. In some instances, there is evidence that engagement with apps can be indicative of real-world spatial behavior. Coutrot et al. (2019) have shown, for example, that wayfinding in apps can serve as a reliable estimator of real-world wayfinding performance. In many instances, trip-making functionality of apps is directly connected to retail information systems and coded aspects of the customer journey: this is particularly evident in mobile recommender systems (Chatzidimitris et al., 2020) and in geo-targeted advertising (Friedrich et al., 2009).

A perhaps increasing number of transactions that walkers make with people and things while walking are now brokered Online. As a consequence, walkers engage with touchpoints that coexist in the tangible world of the transactions, as well as with additional Online channels such as software agents, identification and verification systems, payment systems, database access, content and information retrieval, and cloud storage. Many of these transactions are actively entered into by walkers (e.g., tapping identification to a reader to gain entry to a building, opting to scan a QR code on an advertising display, or geo-tagging and annotating a digital photograph and uploading it to cloud storage). Other transactions while walking are passively generated, as in smart city monitoring systems (Akhter et al., 2019; Kitchin, 2015). However, the significant data science functionality of the retail omnichannel that we just mentioned—automation, linkage, and actionability—is not always easy to tease from traditional forms of urban data. In response to these challenges, many scholars of urban science have turned to multi-channel data acquisition strategies in ways that resemble retailers’ forays into the omnichannel. As we discussed in Section 4.2.5, social media are now a significant component of the retail omnichannel, handling many facets of the customer journey, but particularly advertising and customer service. Similarly, in urban science, a large volume of research has been invested into exploring whether connections can be made between data from social media channels and aspects of urban walking. A key thread in this work connects the content that walkers generate and interact with on social media platforms to geographic references embedded into that content directly, or by association as metadata. The connection between content and location can provide structure to establish geographic context and around the site of content creation, in ways that bridge the media channel space of blog posts (messaging, video-making, photography, annotations such as “likes” or “check-ins”, etc.) with tangible touchpoints along walks, such as entry to establishments, time spent in particular urban space and places, crowding effects of multiple users sharing space and time, and so on. As with data in the retail omnichannel, much of the geography associated to social media content can be automatically generated, using GPS, placename attribution to text, or GeoIP services (Croitoru et al., 2013; He et al., 2015; Kelm et al., 2013). While social media data may not personally identify users, they are generally able to be narrowed uniquely to a given user ID. However, geo-referenced social media produced by commercial services are generally difficult to come by at fine resolution for large numbers of users, or covering wide areas of urban space. Social media companies have these data, but they generally do not share them with the public beyond small sample sets. Additionally, while some aspects of social media content have been shown to be usefully allied to people with accuracy (age demographics in particular), other components of those media have been revealed to have flimsy associations to real people and real things, particularly when considered by geography (Flanagin & Metzger, 2008; Hecht & Stephens, 2014). Of course, retailers expend considerable effort and resources to build coherent omnichannel data science for the customer journey. Their collection methods are usually proprietary and the resulting data products are generally walled within their own commercial ecosystem. Urban scientists cannot feasibly marshal the same level of resources. However, there are perhaps significant lessons to be learned by urban scientists from the omnichannel approach used by retailers to study the customer journey.

### Data granularity

In many aspects of urban science, a general sense has long persisted that data availability is lacking relative to the information that we need to drive the science forward. In areas of urban science that deal fundamentally with individual people (as is the case with walking), data availability is almost a perennial problem. The influx of big data to urban science has helped to alleviate availability challenges somewhat, but thus far these big data are dominated by social media, which can be reasonably viewed as a relatively lackluster indicator of real-world behavior (Lazer et al., 2014), or even wholly unreliable (Jagadish et al., 2014), and perhaps particularly so in urban science (Kitchin, 2014). There is perhaps a convincing argument to be made that big data have not actually helped urban science with meaningful insight. For example, Batty (2013a) has discussed how big data may shift the ideation of urban science toward short-term circumstance at the expense of long-run phenomena. (A related argument is well-made by Barnes (2013)). Scholars in arts and letters have pointed out the obvious: that big data is based on trends in data, which can be a poor substitute for formal scientific inquiry (Ebach et al., 2016: p.3) sourced in long-standing traditions of discovery on carefully reasoned models and hypotheses (ibid. p.4). Realistically, urban science is often better-informed by observational data that are derived from sources (as) close (as possible) to urban phenomena; these data are usually hard to gather and rarely accessible via automated means (Goffmann, 1963, 1971). Atop all of this discussion, one must consider that pedestrian behavior is fundamentally and almost frustratingly individual and complex, and the dynamics of that behavior on high streets produces so much interactivity and knock-on complexity as to be opaque to empirical inquiry (Batty & Torrens, 2001).

One of the impressive features of retail customer journey analysis is the high-detail reach of its data-gathering and analysis capabilities. Examples of the sorts of detail that can be obtained by studying the customer journey include work by Berendes (2019) (with recruited customer participants) to isolate individual high street customer journeys using mobile beacons. Berendes (2019) used allied participant-provided survey data to determine shopper goals, and trajectory locations to infer shopping stage. This approach is not unlike travel diary schemes with GPS data (Shen & Stopher, 2014). Another example is that of Chen et al. (2019), who used eye-tracking hardware to examine shoppers’ view distance in an airport mall. In essence, they were (directly) able to gather data on shoppers’ perceptions of servicescapes.

What is perhaps distinctive about retail customer journey analysis is the ability to tie-in to touchpoints and to the underlying and ambient servicescape. In retailing, customer journeys are routinely traced to individual customer movement trajectories through retail spaces, and the customer journey through decision space often covers large periods of a customer’s shopping history. Retailers are generally well aware of how individual customer journeys nest into larger groupings of customer and shopping typology. Moreover, retailers often have well-devised schemes for mapping individual customer journeys to service blueprints. The collective result of these capabilities is that retailers can build elaborate (and empirically-based) *explanatory context* for individual retail behaviors that they observe. In many retail operations, this context is directly fed to KPIs that yield actionable levers for service adjustment. Work by Berendes et al. (2018) illustrates how context can be built directly from customer journey data. Berendes et al. (2018) presented a markup and modeling language scheme for classifying and contextualizing touchpoint event logs cast from retail high street customer journeys. Although the scheme relies on human input to code the logs, Berendes et al. (2018) demonstrate how the system could possibly be automated.

What lessons can be learned for urban science focused on walking? Walking is, at its core, an exercise in individual agency. Building understanding of that agency requires individual-level data. Without data on individual walkers, it is always going to be challenging to study their behavior in ways that adds to knowledge of their individual agency. Paucity in suitable data can actually constrain the types of analysis that can be built, essentially shoehorning knowledge generation into methods that limit their applicability to the real-world in a sort of self-defeating circle. This issue was discussed by Batty et al. (2003a): “Progress in developing science at this fine scale has been immensely slow for human systems” (p. 674), and much of the reasoning behind this is because, “Data requirements are enormous, always less than optimal, quite unlike aggregate modeling where parsimony is key.” (p. 675). Many analyses (including those under the umbrella of “big data”) attempt to work-around the paucity of individual data by inferring mobility from coarse data (Hong et al., 2017; Brockman et al., 2006; González et al., 2008; Sevtsuk & Ratti, 2010; Guo & Karimi, 2017; Yang et al., 2019; Morris & Zisman, 1962). However, within urban science, the triple challenge of ecological fallacy (Wrigley et al., 1996), modifiable areal unit problems (Openshaw, 1983), and the fallacy of inferring behavior from observed activity (Louviere et al., 2000) make it incredibly difficult to build fine-granularity data from these aggregate data products, no matter how “big” they are in corpus size. Batty et al. (2003a) discussed another challenge, originally raised by Ashby (1958): the “law of requisite variety” (Batty et al., 2003a: 696), which holds that model detail should be matched with equivalent data detail. It would seem, by extension, that one’s information regarding people's behavior should be as up-to-date as the dynamics of that behavior in the systems that you are modeling. These data *are* available in customer journey analytics. It may be interesting to explore the twin questions of how retailers get the data, and how might urban scientists be able to gather similar data? Work in retail geography at micro-sites, which was popular before the big data era, had actually made significant progress toward revealing geographies of mobility at hyper-local urban scales of streetscapes, but research and development in the field seems to have largely tapered off since the end of the 1980s (Brown, 1987, 1988). Perhaps it is time to revisit that research, which was originally formed around case studies, but now using some of the automatic data-gathering schemes that come from customer journey analytics.

### Fast and slow data via Wi-Edge

The speed at which retailers can gather data about their operations is often important in considering how they can leverage the information that the data provides as insight to their business activities. Retail stores have access to a range of “fast” data that is continually replenished as customers walk from high streets through their doors. In a straightforward manner, this includes the counts and demographics of shoppers as they enter, and the duration of their visit before they leave. These data can easily be tallied in real time without loss in fidelity. Over longer periods of time, the fast data can be allied to “slow” in-store data regarding customer visits, e.g., by fusing it to sales receipts to compose counts of return visitors and metrics of customer retention, average value of transaction per visit, purchase volume per customer, value platform of customer, and so on. With additional linkages to Online advertising and shopping systems (through credit card matches to other stores in their network, use of customer loyalty programs, customer scanning of QR codes, etc.), this information may even be extended to cross-channel information.

Big data are often characterized as data with variety, velocity, and volume (Thakuriah et al., 2017), but the velocity of data are not well-considered in many discussions of urban science, which for the most part considers that researchers will go out, collect data, and pore over it for some time thereafter to produce insight. Batty et al. (2003a) discussed this issue with reference to urban modeling and their comments are relevant here to our consideration of data-gathering for pedestrians: “Strictly speaking, with models composed of individuals, there should be data on the decision-making events associated with each individual throughout the time periods and across the space associated with each decision event. What is usually possible is good data on the density of crowds but not on paths taken by individuals.” (p. 676). Ashby (1958) also discussed a related point, regarding informational currency, mentioning that for human systems, the data that might describe the phenomena being considered are quickly out-of-date, which makes it difficult to derive hard-fast rules of behavior for human systems: “Thus the rule “collect truth for truth’s sake” may be justified when the truth is unchanging; but when the system is not completely isolated from its surroundings, and is undergoing secular changes, the collection of truth is futile, for it will not keep.” (p. 93).

Retailers can assemble fast and slow data on customer journeys because they have, essentially, access to long-term observatories on those journeys, through in-store monitoring and information systems, as well as linkages to out-of-store journeys through the omnichannel. How might fast and slow data be built in urban science? Here, we might consider developments in urban computing (Zheng et al., 2014b), which directly consider very fast-incoming streams of data, going as far as to specify new forms of computing such as edge computing and system-on-chip computing that can begin to build analyses on these data as soon as they hit a sensor. We may also consider that pedestrians themselves generate very “fast” data on the devices that they carry when negotiating city life. This is already well-known for location-aware technologies such as GPS and Wi-Fi, which have rapid refresh cycles on the order of ~ 100 Hz. Recent work with inertial measurement units (IMUs), which now commonly feature on phones to support detection of device movement such as picking up calls, has shown that relatively fast data can be generated for assessment of pedestrian navigation. Xing et al. (2017) used IMUs with an artificial neural network to perform pedestrian step detection and stride length at rates of 100 Hz. Chen et al. (2011) used electromyogram (EMG) sensors to detect leg movements of pedestrians and to interpret stride length at 1 kHz. Analysis of these data are usually performed off-line, however. If we consider their potential use for mass observation of pedestrians, currently thorny issues of how to stream the data to centralized information processing infrastructures would need to be addressed. Edge computing (Satyanarayanan, 2017; Taleb et al., 2017), which supports communications of pedestrian sensor data to nearby small factor computing, or even direct sensing on-device (via smart city infrastructure), can support these types of hand-offs. For example, Intel and Nokia Solutions and Networks (2013) introduced the idea of mobile edge computing (MEC). MEC reconsiders communications base stations (such as Wi-Fi access hubs) more broadly as “Radio Applications Cloud Servers” (RACS). RACS may be tasked with performing large computing tasks, with the advantage that the computing sits close to the mobile user and device. Recent work by Potdar and Torrens (2019), for example, has shown that pedestrian movement relative to lighted crossing infrastructure can be detected, in real-time, using computer vision on edge devices with a temporal resolution of ~ 5 times per second in crowded downtown scenes. These types of applications perhaps form a new type of coupled telecommunications and computing infrastructure, through the combination of Wi-Fi and edge computing to form what we could term to be "Wi-Edge", specifically designed to extend the reach of smart functionality to streetscapes. Wi-Edge could be instantiated in many forms. Beckman et al. (2016) introduced the “Waggle” framework for uniting many varied sensor streams on one platform, as part of their vision for the “Array of Things” concept for smart city sensing and computing (Catlett et al., 2017). The Array of Things essentially extends the Internet of Things (Dourish, 2016) toward edge computing. It is also feasible that automobiles and other vehicles might also cast their increasing vision of streetscape scenes to pedestrians, with the potential that large-scale observatories of individual pedestrians in urban context could manifest as a by-product of advanced driver awareness systems (ADAS) and autonomous driving (Massaro et al., 2017; Goodchild, 2018). For example, the *Trellis* system introduced by Qi et al. (2017) has successfully shown that it can estimate ambient pedestrian flow, automatically, from vehicles. In this way, vehicles could serve as mobile carriers for Wi-Edge.

### High street information systems

As mentioned briefly in the beginning of this paper, a relatively recent thread of research and development has emerged around the concept of high street customer journeys and information systems that can dock them to retail operations, which we will refer to hereafter as “customer journey information systems” (CJIS) (Torrens, 2022). Berendes et al. (2018) introduced a conceptual model for featuring key elements of the customer journey on high streets and they described a markup-based modeling language that could be used to map those journey elements to retail operations. That work culminated in what they termed as a “High Street Journey Modeling Language (HSJML)” that can frame online and offline customer journeys with supporting “digital evidence” (p. 221). While Berendes et al. (2018), are apt to point out that the HSJML does not account for decision-making behavior of customers, this missing aspect could, conceivably, be addressed by urban science.

CJIS and HSJML are conceptually parallel to development in urban informatics that propose the idea of “community as a service” (CaaS). CaaS extends the notion of software as a service (SaaS) (Allen et al., 2012) to physical outdoor environments such as streetscapes. Bartelheimer et al. (2018), for example, introduced the idea of community information-sharing platforms for high street retailing, as a potential mechanism for communities of local retailers to build integrated information systems. Schmidt et al. (1999) described how SaaS can be designed to interplay with mobile computing to fashion an “awareness device” (p. 900). Their argument advances the idea that location-aware technologies (such as those used by customers on high streets) may be used to provide context that can be leveraged for computing. A similar argument could be raised, in which we consider that location-aware technologies might *relay* contextual information from the high street to CJIS. This information could come from the pedestrian (indeed, most smart devices routinely guess at users’ identities in various ways) and their use of the device for retail operations (browsing goods, checking prices, consulting reviews, etc.), but also from the sensing capabilities of the device (e.g., motion, movement, optical, sound, proximity, and even biometrics). Much of the context that Schmidt et al. (1999) discussed could easily be used to automatically and tirelessly build understanding of pedestrian journeys, including journey habits, social interaction, tasks, and nearby infrastructure (p. 895). Amaxilatis et al. (2018), for example, introduced a broad platform for pervasive sensing experiments at urban scale that could conceivably provide the information infrastructure to support this.

It is also feasible that CaaS platforms could be integrated with edge computing to directly sense and build fast information streams for urban environments. Satoh (2021) recently introduced a smart digital sign that makes use of very localized context around retail displays to guide the touchpoint information that a customer will encounter in a store during their journey. This context is considered simply, via the presence of radio-frequency identification (RFID) tags within a small distance of a retail touchpoint, but a key innovation in the approach described by Satoh (2021) is that interaction with the RFID is tight-coupled to retail information systems using a series of mobile agents. The agents introduced by Satoh (2021) are simple in specification, designed to run on computationally-light systems merely as brokers to manage touchpoint activity at the retail object “front-end” and the commensurate response at the retail information system “back-end”. Nevertheless, the working online-offline connection between retail operations and the customer journey via physical touchpoints is an incredibly innovative development. A key point in the implementation details of the system provided by Satoh (2021) is that each agent has the capacity to advertise its presence and capabilities and to transfer its code (and state data) from one device to another through the information system (p. 75). It is perhaps straightforward to consider, at least in concept, how similar contextual signs could be developed for outdoor advertising kiosks on high streets, with benefits for high street retailing, but also urban service provisions such as wayfinding, emergency routing, notification of detours, etc.

### Profiling and geodemographics

Geodemographics involves using a set of geographic data science methodologies to build profiles of areas (market segmentations) based on the demographics of their inhabitants (Birkin & Clarke, 1998; Goss, 1995; Longley, 2012). Geodemographics are most popularly used for marketing, to support advertising campaigns, but have traditionally been limited to coarse geographical units. Our review of customer journeys suggests that retailers have extended capabilities for exploration of individual customer profiles that might sit “underneath” coarse geodemographic units. By extension, there would seem to be broad potential for geodemographic profiling at individual level for urban science. Although, we note that the term “profiling” hints at some of the serious privacy concerns that data science of this kind raises.

Burns et al. (2018) introduced a methodology for building individual-level geodemographic profiles from UK Census Small Area Microdata (which are usually deliberately hobbled by geography to protect the privacy of households). Similar schemes for inferring micro-level data from coarse Census sample data were introduced by Blodgett (1998) in the United States, using data-mining. The approach developed by Burns et al. (2018) relies on micro-simulation to essentially synthesize likely individuals from coarse data, using what they term as a “Population Reconstruction Model” (p. 427) based on methods for synthetic data population generation (Harland et al., 2012; Beckman et al., 1996; Smith et al., 2009). Smith (2019) proposed the concept of second-order geodemographics as a by-product of algorithm-driven analysis of “consumer lift” within advertising, i.e., the success in uptake of marketing strategies by consumers. Smith’s (2019) work is relevant to our discussion here because of the connection between consumer lift and visiting patterns of people in retailing spaces. Smith (2019) examined the work of retail data analytics firm, *PlaceIQ*, on data harvested by advertising servers as a component of mobile marketing delivery on smart devices. Smith (2019) showed that significant micro-scale patterns of individual pedestrian activity could be associated with broader geodemographic profiles. Zhang et al. (2020) considered the potential for building micro-scale geodemographics from transit card data. They introduced a scheme for creating two-dimensional maps/images of the space-time geography of *individual’s* smart card data (via *Oyster card* databases), which they then subjected to CNN analysis to make predictions of those individual’s demographics. These demographics could then be combined with estimates of the individual’s location to essentially build individual-level geodemographic profiles, directly from smart card data. This work is very relevant to the discussion in our paper, as it directly links touchpoint data (smart card tap-ins) to geodemographic profiling in an automated scheme.

### Controlled experimentation

One of the benefits of the customer journey concept is its ability to support design of store and product layouts with empirical information about likely returns to service efficiency. Retailers can, for example, design new customer experiences and test their impact on customer journeys, often using well-structured KPIs (Underhill, 2009). The same sorts of functionality could be incredibly useful for urban management, design, and planning. Researchers in physics, who have long maintained an interest in crowd dynamics as an example of complex adaptive systems, have established quite sophisticated controlled experiments to collect data on moving pedestrians, particularly within the confines of fixed infrastructure and subject to varying scenarios of emergency movement behavior (Helbing et al., 2007; Johansson et al., 2008; Moussaïd et al., 2009). Feng et al. (2020) make a valid point in raising the issue that students are over-represented in many of these physical experiments because much of the work takes place on university campuses using participant populations at those institutions; similarly, they argue that the temporary approximations of built environment that are erected for these experiments are far from realistic in inducing commensurate behavior (p. 7).

To our knowledge, no controlled physical experiments pertaining to high streets have been established. However, virtual reality environments (VREs) and virtual geographic environments (VGEs) (VREs with mappings to real-world geographies) are increasingly being used to reach wide areas of streetscape for experimental studies. In particular, researchers have employed VR to ease some of the difficulties in establishing controlled physical experiments with pedestrians on pseudo-streetscapes (Mól et al., 2008; Pelechano et al., 2008). We are aware of one example in customer journey analysis for retailing: Mathieu et al. (2011) developed the “*Format-Store*” first-person gaming-type environment to help train shop staff. The environment contains virtual stores, virtual products, and virtual customers. This suggests that VREs and VGEs could perhaps be usefully employed to evaluate customer journeys on retail high streets, and that the customer journey framework could be used alongside urban VGEs to explore pedestrian behavior in simulated city settings. Torrens and Gu (2021), for example, introduced a *Unity*-based immersive VGE for examining how human participants interacted on simulated representations of streetscapes in Brooklyn, NY. However, there is an ongoing debate, particularly in the field of psychology, as to whether virtual reality experiments are a meaningful approximation of real-world scenarios (Blascovich et al., 2002; Loomis et al., 1999; Thompson et al., 2004). Feng et al. (2020) also raise the issue that the technology can cause dizziness (and nausea) in a significant proportion of potential participants, particularly in key populations for retail high streets such as the elderly (p. 7). To our knowledge, there is no work published that demonstrates the use of VR models of retail high streets, although commercial interest in the use of augmented reality and mixed reality is incredibly high (Katwala, 2018).

### Privacy and privacy protection

One must ultimately consider that retail-driven frameworks designed to plan for and to manage (and perhaps to manipulate) moving customers could be quite problematic in transfer to urban science applications, and this starts to get unacceptably invasive if we consider mappings to individuals. This is particularly relevant when we consider that, unlike indoor shoppers who understand that they are entering private spaces, pedestrians cannot (and ought not feel like they should) avoid visiting public urban spaces and that city-dwellers cannot feasibly “opt out” of city life. Retailers’ impressive detail in insight, of course, comes from considerable investigative encroachment into customer’s day-to-day activity in retail environments and with retail information systems. The schemes by which customers might decide to opt-in to those data-gathering systems are controversial, largely because the intent behind the data acquisition is not always readily transparent. For example, customers may be aware that closed circuit television (CCTV) cameras are deployed in stores to prevent theft, but they may be unaware that the same video feeds can be used to track and classify their shopping behavior (so-termed “function creep” (Koops, 2021) from one use scenario to another).

The question of whether and how technologies that drive customer journey analysis might carry over into urban science is potentially quite critical when we consider that urban streets are not stores: pedestrians in public may be quite resistant to the intrusive use of technology that scans, tracks, analyses, and builds inference on their behavior in public settings. The issue of privacy and surveillance of pedestrians is complicated. Legally, pedestrians, moving as they usually do along public streetscapes, generally have no expectation of privacy in those settings. At the same time, pedestrians may not have not opted-in to surveillance and into the (many) uses that information about their activities might be put, which we have now pointed out may be individualized across a bewildering array of means. That observation of pedestrian activity produces individual data, then, raises questions of pedestrians’ rights of access to and protection of data procured from their time on high streets. This balance of expectations of behavioral privacy and rights to be informed of behavioral data is part of an ongoing debate in urban science about digitization of the public realm (Curry, 1997). That debate takes on many forms of concern, notably about the emergence of digital places (Curry, 2008; Zook et al., 2004) and their societal ramifications (Poom et al., 2020). There is an obvious unease that one might feel when thinking that technologies designed for retail intelligence in indoor private retail establishments might creep into public spaces. Anybody that does not want to be subject to in-store retail surveillance can simply choose not to shop in a given store. However, avoiding what Pal and Crowcroft (2019) called “surveillance capitalism” when that intelligence is trained on public space is much more difficult. We need to be mindful, then, that any (commercial) developments in retail intelligence potentially feed into an evolving “automated geography” (Dobson, 1983; Graham, 2005; Thrift & French, 2002) with the potential to improve urban life through efficiency and transparency (Townsend, 2013), but also with a pervasive reach into the erosion of identity (Dobson, 2009), governance (Kitchin, 2020), and civil liberty, and the potential to exacerbate existing forms of data-based and algorithmic bias (Coletta & Kitchin, 2017) in the treatment of people in the spaces that they call home (Gabrys, 2014).

Retailers have varied (but mostly financial) motivations for collecting high-detail data about pedestrians as they engage in the customer journey, and there are justified concerns about the forms of retail surveillance that might spring from their activities (Cantrell, Ganz, et al., 2015; Clarke, 1994; Elnahla & Neilson, 2021). How retailers’ motivations sit with the public is somewhat murky territory. As Farshidi (2016) and Nguyen (2018) discussed, relative to legal standards, these activities may well violate privacy norms, but are often legal. However, retailers are also motivated to *protect* the privacy of the data that they collect, because they have legal obligations to do so in some jurisdictions, and because data breaches are bad for business (Martin et al., 2020). Pizzi and Scarpi (2020) showed that customers’ sentiment about their data privacy can influence retail patronage directly. Inman and Nikolova (2017), for example, have shown that customers often evaluate the privacy implications of new retailing technologies when considering whether to adopt them. Marriott et al. (2017) came to the same conclusions with respect to m-commerce in particular.

Many of the data sources from social media and other Web 2.0 platforms that we discussed have long been recognized as wide open to individual, collective, and social privacy concerns (Elwood & Leszczynski, 2011). Indeed, one of the central principles of geodemographics—as pioneered for retailing (Goss, 1995)—has been to associate individuals with algorithmic classifications polled from the context of their location, or so-called “neighborhood targeting” (Harris et al., 2005). As we mentioned in Section 5.5, geodemographic targeting is now being “miniaturized”, i.e., applied to finer-resolutions of insight and specificity (Burns et al., 2018), at the same time that it is becoming more dynamic. New geodemographic schemes, for example, have been developed specifically for tap-in data (Zhang et al., 2020). This creates something of a privacy dilemma, if one considers that activities by retailers to protect customers’ privacy can sometimes become moot when the customer then goes on to advertise their location, identity, activity, and preferences on social media.

We must also perhaps recognize that high street retailing is now firmly embedded within the smart city more generally (Hollands, 2008; Pantano & Timmermans, 2014; Pretz, 2019). Kitchin (2015), for example, has long advocated for realization that smart cities, perhaps by design, are replete with geosurveillance. Work in GIS, in particular, suggests some potential paths of solution to the privacy challenge. The traditional approach, developed initially in GIS for privacy masking (Kwan et al., 2004), has been to obfuscate location data that is delivered to LBS. The *PROBE* scheme developed by Damiani et al. (2009), for example, combines obfuscation with privacy measures that may be defined in policies by the user. Research by Nergiz et al. (2008) has shown that this can also work for trajectory anonymization. Early work in obfuscation analyses in GIS essentially reached the same conclusions, e.g., see Kwan et al. (2004). Authors such as Duckham and Kulik (2005) have argued that obfuscation to essentially “muddy” location-specific data is probably *integral* to LBS, and that attention could usefully turn instead to negotiating how *much* privacy users are comfortable in ceding.

However, from the perspective of retailers, there is perhaps little motivation to obfuscate incoming data, as it also obfuscates their insight. For customer journey analysis, in particular, it is usually desirable that retail insight stretch down to the individual. Indeed, much of what we have reviewed in this paper has shown the tremendous sophistication with which individual-level detail can be built from retail data science. Recent approaches in spatial analysis have shown that some level of data detail can be retained when spatial statistics and geostatistics are applied to geographic information specifically to mask identifying information and locations. Wang and McArthur (2018) and Gao, Janowicz, et al. (2013), for example, have shown this for trajectory data. Oksanen et al. (2015) introduced a modification to kernel density estimation that preserves privacy in geostatistical surfaces by, essentially, masking the underlying count statistics. They applied the technique to coarse mobility data from fitness tracking archives. Andrienko et al. (2009) discussed how *spatio-temporal* privacy might be achieved for movement data using spatial analysis.

There have been various angles of approach to the privacy problem of smart cities (Curzon et al., 2019) from computer science, some of which have bearing on the privacy concerns raised in this paper and may suggest further paths for addressing privacy on the customer journey. Work in edge computing, in particular, has attempted to address privacy concerns. The edge approach, and particularly the new forms of sensor-adjacent artificial intelligence (“edge AI”) that it supports (Woods, 2018), is rapidly being considered by retailers as a way to efficiently collect data on the customer journey. Considerations for edge-based privacy-by-design address the idea that privacy-preserving or privacy-conscious computing could be performed by first principles at the edge, before data are streamed to centralized computing resources where they may be archived and linked to other identifying data. This is perhaps something of a perennial challenge, as sensors and algorithms for processing street-side data are continually advancing, currently much faster than privacy measures to tackle them can keep up with, just as laws and regulations struggle to match pace (Farshidi, 2016). In concept, however, one idea might be to permit detailed data analysis locally, perhaps at the individual store, while masking those data before they pass on to centralized systems, where they would otherwise be vulnerable to function creep. The idea for “micro-aggregation” of location data such that it is cloistered to hyper-local settings suggests some pathways for accomplishing this in GIS by essentially generating synthetic data with properties similar to the original data (Domingo-Ferrer et al., 2010). In computing, this aligns with schemes for “k-anonymity” (Sweeney, 2002), i.e., churning identifiable data into still-usable aggregates such that individuality is masked; and, in geographic terms (Shokri et al., 2011), the spatial attributes of the data remain. From computer science perspectives, there are emerging ideas that privacy issues may be resolvable directly in machine-learning. Fitwi et al. (2021) have proposed that CNNs, embedded within SaaS, could be deployed to edge computing *systems* to both detect privacy-eroding data and to mask those data before they move from the edge to back-end computing infrastructure (so-called end-to-end systems).

Other approaches are designed to address privacy in the network infrastructure of smart cities, i.e., to catch privacy-eroding data as it is communicated. This is an interesting approach, as it opens up the possibility that privacy-preserving network *protocols* could be developed, with the further implication that these could possibly work in tandem with general communications protocols that underpin most ICTs for LBS. For example, Lu et al. (2014) introduced a querying privacy framework specifically for LBS-based mobile social networks that bypasses the need for privacy measures (such as tokens) on a centralized server (unlike, for example, the centralized *CacheCloak* scheme for LBS developed by Meyerowitz and Choudhury (2009)). Jiang et al. (2019) recently introduced dedicated “location protocols” that act in a privacy-preserving fashion. They focused on localization in wireless systems, applying privacy schemes directly to the location-estimating algorithms that operate on trilateration and multi-lateration. This is significant, as it masks the actual location of the user, as their position is actively being calculated within the wireless system, at an algorithmic/heuristic level.

## Conclusions

In this review paper, we have explored an idea from retailing—*the customer journey*—as a framework for the study of walking in urban science, as well as a concept for advancing retailers’ ability to analyze the customer journey outdoors, on ambient streetscapes around their stores, and the activity of pedestrian shoppers that they support. Our main argument is that the customer journey framework could serve as a useful individual-scale scheme for building urban science around individual pedestrian experiences on streets. Our consideration of this idea comes primarily from the perspective of urban informatics: the information that can be gathered in service of understanding cities by examining individual pedestrians as embodied in moment-to-moment settings of urban life. Our use of an information lens is motivated by the increasing range of high-resolution insight available to retailers about customer behavior in indoor and outdoor shopping environments, due in large part to emerging novelty in data science and sensing. In many ways, the ubiquity of information that retailers maintain about customers stands in some contrast to the relative paucity of empirical ground truth available to urban scientists to theorize with. Our review has focused on how *data* that are produced by customer journeys along retail high streets and through urban streetscapes might inform pedestrian studies. These data, we argue, are of particular relevance to urban science because they are “human cast”, cast by pedestrians as they experience urban settings, but they are also interpreted by their evolving context relative to sites and facilities. In this way, then, customer journeys present opportunities for urban scientists to look at the experience of walking through the two-way lens of the walker and that of the ambient built and social environments of the streetscapes that they move through.

The opportunity for considering customer journey frameworks for urban data science stems in large part from the extensibility and resilience of the customer journey concept against a backdrop of continual advancement in sensing, computing, and inference technologies that have swept through retailing operations. In particular, the steady increase in the volume, pace, resolution, and reach of retail data collection mirrors that of urban data science in some critical areas of overlap. In this review, we examined existing (but perhaps mostly unintended) synergies between retail ideas of the customer journey and urban informatics for pedestrian surveys, movement tracking, tap-ins to transit systems, data-mining of activities and actions, and computer vision. We also outlined a set of avenues for future possible dedicated research for urban science around the customer journey frame. These include investigation of potential urban omnichannels, data science approaches to granularity, new computational schemes to reconcile fast and slow data, potential extensions of customer journey information systems into broader high street information systems, shifts in the resolution of profiling and geodemographics to individual scale, and the broad potential for experimentation through controlled tinkering in simulations, virtual reality, and virtual geographic environments.

The issue of privacy permeates almost every aspect of this discussion. Many of the same technologies that underpin retail data science and computing are now being used (or considered for use) in city-oriented data science. It therefore seems reasonable that we should consider that the surveillance architectures from retailing might straightforwardly find their way into urban informatics. We must acknowledge that there is a huge risk inherent in the function creep of transferring data-driven commercial value propositions of retail analytics, unfiltered, into urban science that is usually focused on *society-facing value propositions*. Design of any of the possible research that we have suggested here must surely also consider how privacy (and security) can be baked-into the systems, by designs that counter their ability to intrude on individuals.

## Data Availability

Data sharing is not applicable to this article as no datasets were generated or analyzed during the current study.
